# Kaposi’s sarcoma-associated herpesvirus processivity factor (PF-8) recruits cellular E3 ubiquitin ligase CHFR to promote PARP1 degradation and lytic replication

**DOI:** 10.1371/journal.ppat.1009261

**Published:** 2021-01-28

**Authors:** Woo-Chang Chung, Seungrae Lee, Yejin Kim, Jong Bok Seo, Moon Jung Song

**Affiliations:** 1 Virus-Host Interactions Laboratory, Department of Biotechnology, College of Life Sciences and Biotechnology, Korea University, Seoul, Republic of Korea; 2 Metabolome Analysis Team, Korea Basic Science Institute, Seoul, Republic of Korea; Florida State University, UNITED STATES

## Abstract

Kaposi’s sarcoma–associated herpesvirus (KSHV), which belongs to the gammaherpesvirus subfamily, is associated with the pathogenesis of various tumors. Nuclear enzyme poly(ADP-ribose) polymerase 1 (PARP1) catalyzes the polymerization of ADP-ribose units on target proteins. In KSHV-infected cells, PARP1 inhibits *r*eplication and *t*ranscription *a*ctivator (RTA), a molecular switch that initiates lytic replication, through direct interaction. Thus, for efficient replication, KSHV has to overcome the molecular barrier in the form of PARP1. Previously, we have demonstrated that KSHV downregulates the expression of PARP1 through PF-8, a viral processivity factor. PF-8 induces ubiquitin–proteasome system–mediated degradation of PARP1 via direct physical association and enhances RTA transactivation activity. Here, we showed that dimerization domains of PF-8 are crucial not only for PARP1 interaction and degradation but also for enhancement of the RTA transactivation activity. PF-8 recruited CHFR for the PARP1 degradation. A knockdown of CHFR attenuated the PF-8–induced PARP1 degradation and enhancement of the RTA transactivation activity, leading to reduced KSHV lytic replication. These findings reveal a mechanism by which KSHV PF-8 recruits a cellular E3 ligase to curtail the inhibitory effect of PARP1 on KSHV lytic replication.

## Author summary

Kaposi’s sarcoma–associated herpesvirus (KSHV), a member of the gammaherpesvirus subfamily, is associated with the pathogenesis of various tumors. Poly(ADP-ribose) polymerase 1 (PARP1), which is involved in various cellular functions, restricts lytic replication of oncogenic gammaherpesviruses by inhibiting *r*eplication and *t*ranscription *a*ctivator (RTA), a molecular switch that activates the viral lytic replication. To abrogate the inhibitory effect of PARP1, reactivated KSHV promotes PARP1 degradation via direct interaction between PARP1 and PF-8, a viral processivity factor. Dimerization domains of PF-8 were found to be critical for PARP1 interaction and degradation and for enhancing the RTA transactivation activity. Furthermore, we found that CHFR, an E3 ubiquitin ligase, is required for PF-8–induced PARP1 degradation and efficient lytic replication of KSHV. This is the first study to show the role of CHFR in viral replication or pathogenicity. This study revealed a molecular mechanism via which gammaherpesviruses overcome the PARP1-mediated inhibitory effect on viral replication: by means of PF-8, which recruits a cellular E3 ubiquitin ligase.

## Introduction

Human gammaherpesviruses, including the Epstein–Barr virus and Kaposi’s sarcoma–associated herpesvirus (KSHV), which mainly establish a latent infection in lymphocytes, are associated with the pathogenesis of various tumors and proliferative diseases. The latent KSHV infection is associated with all types of Kaposi’s sarcoma, primary effusion lymphoma, and multicentric Castleman’s disease [1,2]. In addition to the KSHV latent infection, the reactivation of latent KSHV and lytic replication are critical for virus propagation and spread. The viral reactivation replenishes a pool of latently infected cells and contributes to tumorigenesis. The population of episome-harboring cells diminishes during cell division if the latent virus is not periodically reactivated [3,4]. Therefore, the inhibition of KSHV lytic replication is important for the control of viral infection and tumorigenesis.

Poly(ADP-ribose) polymerase 1 (PARP1) is a nuclear enzyme that catalyzes the polymerization of ADP-ribose monomers (derived from nicotinamide adenine dinucleotide; NAD^+^) on a target protein. PARP1 is involved in various cellular processes, such as the DNA damage response, cell death, chromatin remodeling, transcription regulation, inflammation, and tumorigenesis [5]. In KSHV-infected cells, PARP1 catalyzes the poly(ADP-ribosyl)ation (PARylation) of *r*eplication and *t*ranscription *a*ctivator (RTA), a molecular switch of lytic replication through direct interaction, which inhibits the RTA activity and consequently suppresses lytic replication [6,7]. Additionally, PARP1 modulates DNA replication of KSHV [8,9].

Previously, we have reported a strategy that the virus uses to overcome the inhibitory effect of PARP1 during KSHV lytic replication [10]. The reactivation of KSHV results in PARP1 downregulation. The direct interaction between PF-8, a viral processivity factor encoded by the KSHV *orf59* gene, and PARP1 causes ubiquitin–proteasome system (UPS)-dependent degradation of PARP1. The PF-8–mediated PARP1 degradation enhances the RTA transactivation activity and promotes lytic replication [10]. Nonetheless, the mechanism underlying the PF-8–induced PARP1 degradation has not been elucidated. PF-8 does not contain any known motif that mediates protein degradation. In this study, we mapped the critical domains involved in the interaction between PF-8 and PARP1. Furthermore, a cellular E3 ubiquitin ligase recruited by PF-8 for the PARP1 degradation was identified. Our work elucidates the mechanism through which the virus overcomes the host barrier against efficient lytic replication, which involves hijacking the cellular UPS.

## Results

### PF-8–induced PARP1 degradation through K48-mediated poly-ubiquitination

**Fig 1:**
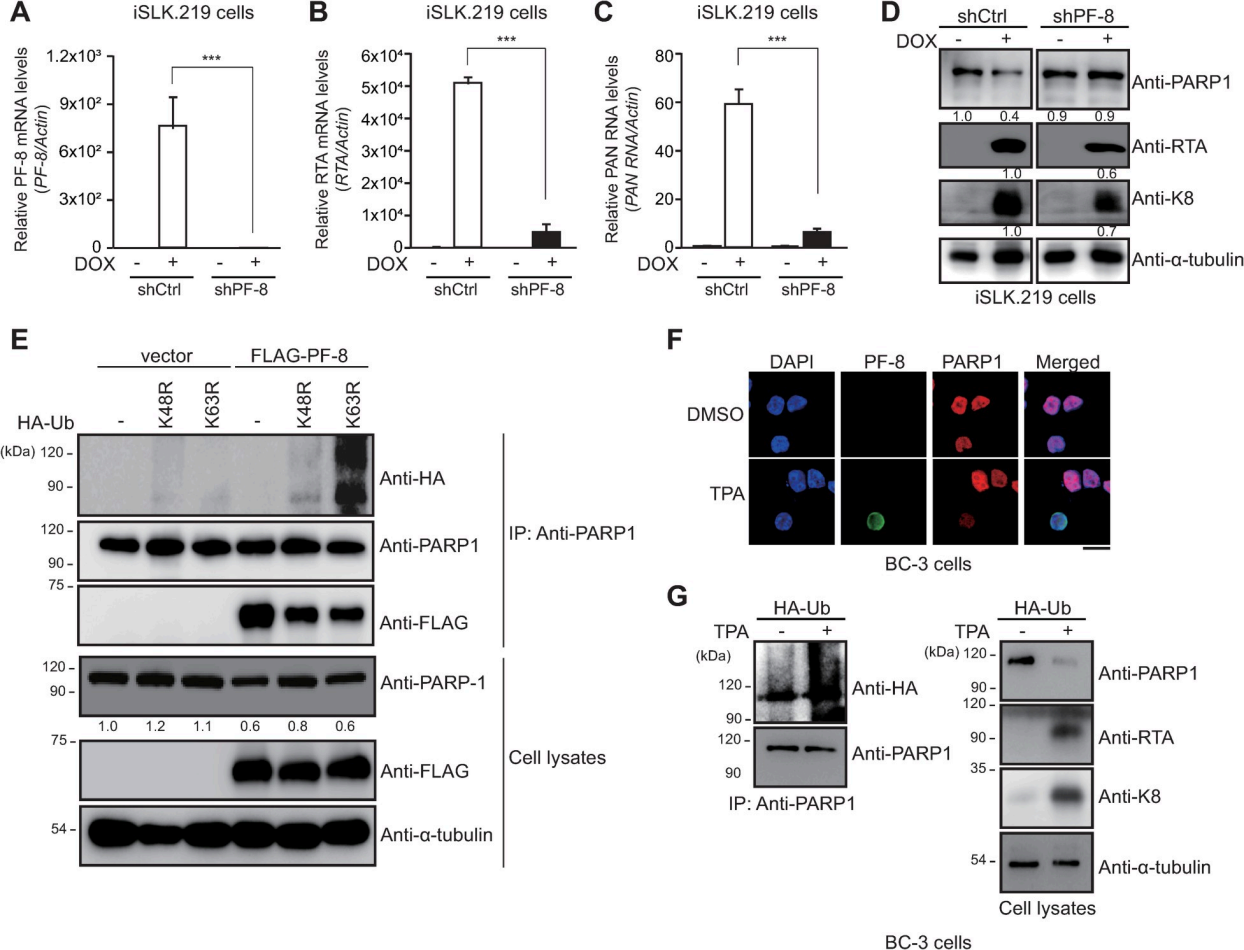
PF-8 induces K48-dependent-poly-ubiquitination–mediated degradation of PARP1 through interaction with the central domain of PARP1. (A to D) PF-8 knockdown iSLK.219 cells and control iSLK.219 cells were generated by transduction of shRNA-PF-8–expressing and control short hairpin RNA (shRNA)-expressing lentiviral constructs, respectively. The expression levels of the PF-8 transcript (A), *r*eplication and *t*ranscription *a*ctivator (RTA) transcript (B) and polyadenylated nuclear (PAN) RNA transcript (C) were analyzed by quantitative real-time polymerase chain reaction (qRT-PCR) after doxycycline (DOX) treatment for 48 h. Statistical analysis was performed by Student’s *t* test (***P < 0.005). (D) The lysates of shCtrl-transfected and shPF-8–transfected cells were subjected to western blotting with the anti-RTA, anti-K8, and anti-α-tubulin antibodies. The expression levels of PARP1, RTA, or K8 relative to those of α-tubulin are indicated. (E) HEK293T cells were transfected with FLAG-tagged PF-8 and hemagglutinin (HA)-tagged UbK48R or HA-tagged UbK63R constructs. The transfected cells were harvested at 48 h post-transfection and subjected to an IP assay with the anti-PARP1 antibody. The cell lysates were studied by western blotting with the anti-PARP1, anti-FLAG-M2, anti-HA, and anti-α-tubulin antibodies. The expression levels of PARP1 in comparison with those of α-tubulin are presented. (F) Subcellular localization of PARP1 and PF-8 in KSHV replicating BC-3 cells. BC-3 cells were treated with TPA, fixed at 24 h post-treatment, and immunostained with anti-PF-8 (green) and anti-PARP1 (red) antibodies. The nuclei were stained with 4′,6-diamino-2-phenylindole (DAPI) (blue). The samples were examined under the confocal laser scanning microscope. Scale bar, 20 μm. (G) Polyubiquitination of PARP1 upon KSHV reactivation. BC-3 cells were transfected with hemagglutinin (HA)-tagged ubiquitin (Ub) constructs. The transfected BC-3 cells were harvested after 12-O-tetradecanoylphorbol-13-acetate (TPA) treatment for 24 h and subjected to an immunoprecipitation (IP) assay with the anti-PARP1 antibody. The cell lysates were studied by western blotting with the anti-PARP1, anti-HA, anti-RTA, anti-K8, and anti-α-tubulin antibodies.

Previously, we have demonstrated that PF-8, a processivity factor of KSHV, induces UPS-dependent degradation of PARP1 via a direct association upon reactivation of latently infected B cells [10]. In the present study, the iSLK.219 cell line, a subclone of iSLK cells that are latently infected with recombinant KSHV.219, was used. iSLK.219 cells emit a green fluorescent protein (GFP) signal during latency and a red-fluorescent-protein signal upon doxycycline (DOX)-induced reactivation of the virus [11]. When PF-8 was knocked down in iSLK.219 cells (Fig 1A), PARP1 levels did not diminish, whereas the expression of viral lytic genes including RTA, PAN RNA and K8, decreased, indicating that PF-8 is necessary to degrade PARP1 and promote viral reactivation (Fig 1A–1D). Results of a PARP1 immunoprecipitation (IP) assay in PF-8–transfected cells revealed that endogenous PARP1 interacted with PF-8, which promoted the degradation of PARP1 through K48-mediated poly-ubiquitination (Fig 1E). In KSHV replicating BC-3 cells, PARP1 was also degraded and co-localized with PF-8 in the nucleus (Fig 1F). KSHV reactivation decreased the PARP1 protein level in BC-3 cells via inducing PARP1 polyubiquitination in BC-3 cells (Fig 1G). These data indicated that PF-8 promotes PARP1 degradation through a ubiquitination-dependent mechanism and consequently facilitates viral lytic replication.

### Identification of PARP1 domain required for PF-8 interaction

**Fig 2:**
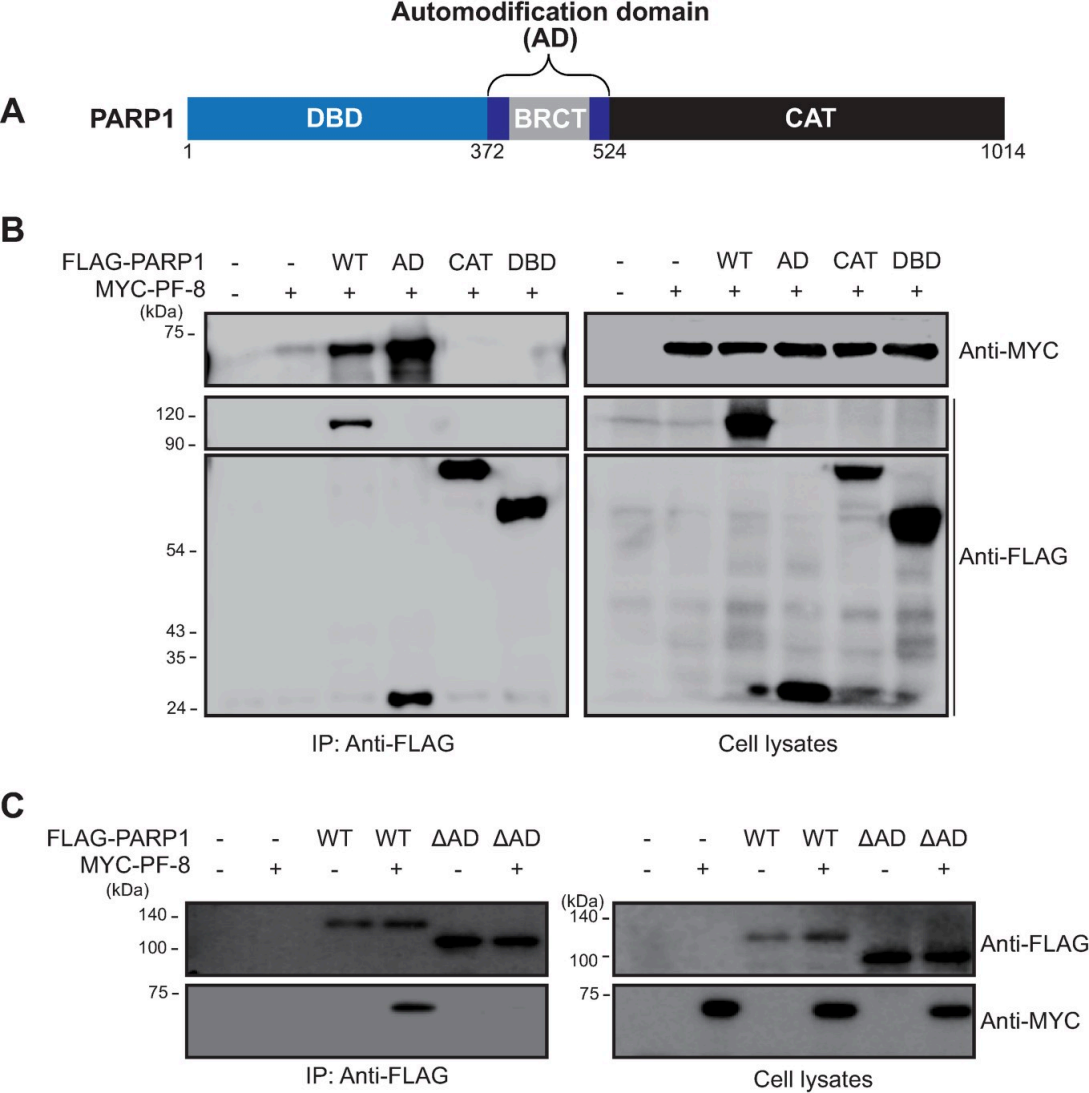
PF-8 interacts with PARP1 through the automodification domain. (A) A schematic diagram of PARP1 functional domains; DNA binding domain (DBD), automodification domain (AD) including BRCT domain and catalytic domain (CAT). (B) HEK293T cells were transfected with plasmids expressing MYC-tagged PF-8 and FLAG-tagged mutants of PARP1. The transfected cells were harvested at 48 h post-transfection and subjected to an IP assay with the anti-FLAG antibody. The cell lysates were investigated by western blotting with the anti-MYC, anti-FLAG-M2, and anti-α-tubulin antibodies. (C) HEK293T cells were transfected with plasmids expressing FLAG-tagged PARP1 or PARP1 ΔAD in addition to MYC-tagged PF-8. The transfected cells were harvested at 48 h post-transfection and subjected to an IP assay with the anti-FLAG antibody. The cell lysates were investigated by western blotting with the anti-MYC, anti-FLAG-M2, and anti-α-tubulin antibodies.

To identify the domain involved in the PARP1–PF-8 interaction, we conducted experiments with PARP1 domain mutants. PARP1 comprises the following three domains: a DNA-binding domain, an automodification domain (AD) with a BRCA1 C-terminus (BRCT) motif (which mediates auto-PARylation and protein–protein interactions), and a catalytic domain for PARylation (CAT; Fig 2A) [12–14]. HEK293T cells were transfected with FLAG-tagged PARP1 domain mutants and MYC-tagged PF-8 to analyze the protein interactions by the IP assay. PF-8 coimmunoprecipitated with full-length PARP1 and the AD, not with DBD or CAT (Fig 2B). In addition, a PARP1 mutant with AD deletion (ΔAD) failed to interact with PF-8, indicating that the AD of PARP1 is necessary and sufficient in the association of PF-8 with PARP1 (Fig 2C).

### Mapping the PF-8 domains critical for PARP1 interaction and degradation

**Fig 3:**
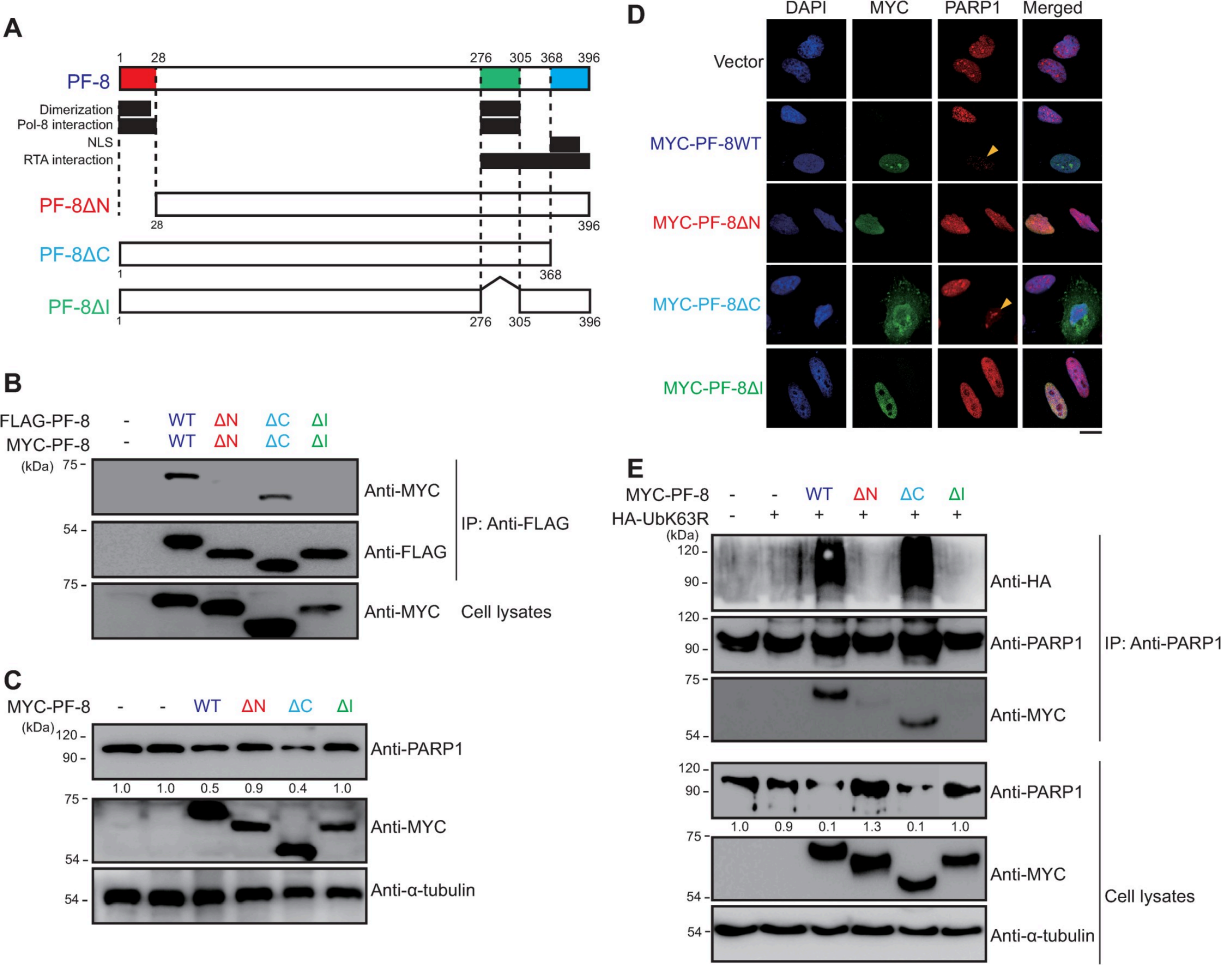
Identification of PF-8 domains critical for PARP1 interaction and degradation. (A) The schematic diagram of PF-8 and its mutants used in this study. (B) Domains of PF-8 critical for its dimerization. HEK293T cells were transfected with FLAG-tagged and MYC-tagged PF-8 mutants. The transfected cells were harvested at 48 h post-transfection and subjected to an immunoprecipitation (IP) assay with the anti-FLAG-M2 antibody. The cell lysates were analyzed by western blotting with the anti-FLAG-M2 and anti-MYC antibodies. (C and D) Domains of PF-8 essential for PARP1 degradation. (C) HEK293T cells were transfected with the MYC-PF-8 mutant constructs. The transfected cells were harvested at 48 h post-transfection and analyzed by western blotting with the anti-PARP1, anti-MYC, and anti-α-tubulin antibodies. The expression levels of PARP1 relative to those of α-tubulin are indicated. (D) HeLa cells were transfected with the MYC-PF-8 mutant constructs, fixed at 48 h post-transfection, and immunostained with anti-MYC (green) and anti-PARP1 (red) antibodies. The nuclei were stained with DAPI (blue). The samples were examined under the confocal laser scanning microscope. Scale bar, 20 μm. (E) Domains of PF-8 essential for PARP1 poly-ubiquitination. HEK293T cells were transfected with the MYC-tagged PF-8 mutant and hemagglutinin (HA)-tagged UbK63R constructs. The transfected cells were harvested at 48 h post-transfection and assayed by IP with the anti-PARP1 antibody. The cell lysates were subjected to western blotting with the anti-PARP1, anti-MYC, anti-HA and anti-α-tubulin antibodies. The expression levels of PARP1 in comparison with those of α-tubulin are presented.

To map the domain of PF-8 that mediates the PARP1 interaction and subsequent PARP1 degradation, we constructed the following domain deletion mutants: PF-8 ΔN, PF-8 ΔI, and PF-8 ΔC (Fig 3A). Some studies suggest that mutant proteins PF-8 ΔN and PF-8 ΔI lack the domains necessary for PF-8 dimerization (amino acid residues [aa] 1–23 and 277–304) and for viral DNA polymerase interaction (aa 1–27 and 277–304) [15–19], and that the PF-8 ΔC mutant protein lacks the region of the nuclear localization signal (aa 369–377) and an unstructured motif [16]. Consistent with the results of previous studies, the PF-8 ΔN and PF-8 ΔI mutants, but not PF-8 ΔC mutant, did not dimerize in HEK293T cells (Fig 3B). Although PF-8 ΔN and PF-8 ΔI showed nuclear localization, the degradation of PARP1 was not observed in either the PF-8 ΔN–transfected or PF-8 ΔI–transfected HEK293T cells and HeLa cells (Fig 3C and 3D). In contrast, the degradation of PARP1 was observed in the PF-8 ΔC–transfected cells (Fig 3C and 3D). The change in the subcellular localization from the nucleus alone to both the nucleus and the cytosol after PF-8 was truncated (the PF-8 ΔC mutant) indicated that the deletion of 26 aa from the C terminus of PF-8 was not sufficient to block the nuclear transport of this protein, in contradiction to the results of another study (Fig 3D) [16]. The degradation, interaction, and poly-ubiquitination of PARP1 were defective in the PF-8 ΔN–transfected and PF-8 ΔI–transfected HEK293T cells, whereas these characteristics were similar between the PF-8 ΔC–transfected and wild-type (WT) PF-8–transfected HEK293T cells (Fig 3E). These findings suggest that the domains participating in PF-8 dimerization are essential for the PARP1 interaction and ubiquitination-dependent degradation.

### Dimerization domains of PF-8 are essential for the enhancement of RTA transactivation

**Fig 4:**
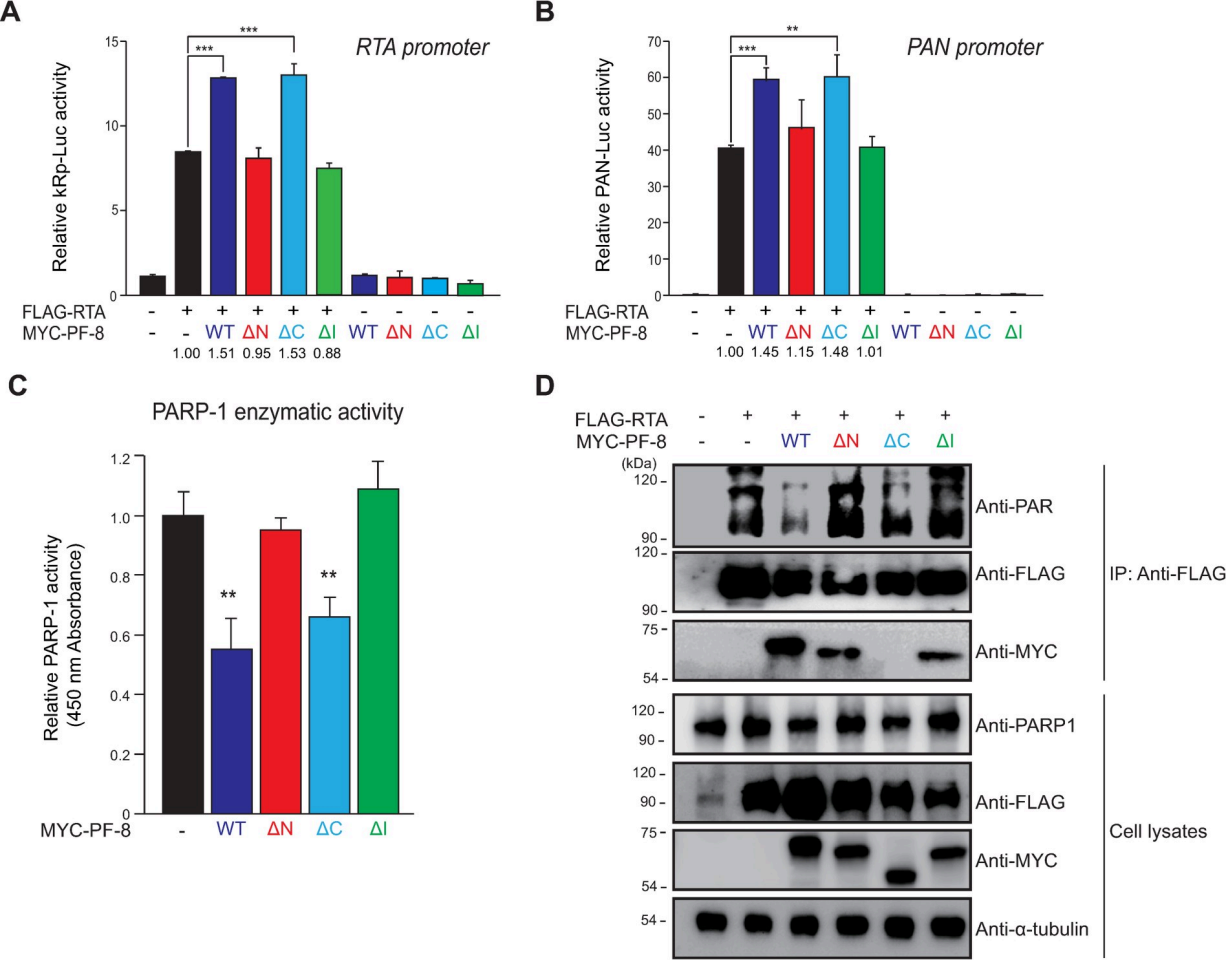
Binding of PARP1 to PF-8, not RTA, is essential for PF-8–induced RTA transactivation enhancement. The latter is mediated by decreased levels of poly(ADP-ribosyl)ated (PARylated) RTA. (A and B) Luciferase reporter assays of PF-8 mutants. HEK293T cells were transfected with reporter construct pGL3-kRP-Luc (A) or pGL3-PAN-Luc (B) (300 ng) and MYC-PF-8 mutants (150 ng) in the presence or absence of the FLAG-tagged RTA expression plasmid (25 ng). The cells were harvested at 48 h post-transfection for luciferase reporter assays. Each transfection was performed in triplicate, and the EGFP-expressing plasmid served as an internal control. The increased fold values of promoter activity relative to the RTA alone sample are indicated. Statistical analysis was performed by Student’s *t* test (**P < 0.01 and ***P < 0.005). (C) PARP1 activity in the cells expressing PF-8 mutants. HEK293T cells were transfected with MYC-tagged PF-8 mutants. The transfected cells were harvested at 48 h post-transfection. The PARP1 inhibition activity in 50 μg of cell lysates was analyzed using the PARP1 assay kit with histone-coated strip wells at 450 nm absorbance. Statistical analysis was performed by Student’s *t* test (**P < 0.01). (D) PF-8 mutant–mediated PARylation of RTA. HEK293T cells were transfected with MYC-tagged PF-8 mutants and FLAG-tagged RTA constructs. The transfected cells were harvested at 48 h post-transfection and subjected to an immunoprecipitation assay with the anti-FLAG-M2 antibody. The cell lysates were investigated by western blotting with the anti-PAR, anti-PARP1, anti-FLAG-M2 anti-MYC, and anti-α-tubulin antibodies.

PF-8–mediated PARP1 degradation is reported to promote the RTA transactivation activity [10]. Therefore, we set to determine the PF-8 domain that is involved in the enhancement of the RTA transactivation activity. In reporter assays with RTA (*kRp-luc*) and polyadenylated nuclear (PAN) RNA (*pPAN-luc*) promoters [20,21], mutants MYC-PF-8 ΔN and MYC-PF-8 ΔI did not enhance RTA-mediated transactivation (Fig 4A and 4B). On the contrary, the RTA-mediated transactivation activities in the MYC-PF8 ΔC–transfected cells were similar to those in the MYC-PF8 WT–transfected cells. The impact of PF-8–induced PARP1 degradation on the enzymatic activity of PARP1 was evaluated by means of the HEK293T cells expressing either WT or mutant PF-8 (Fig 4C). The activity of PARP1 was lower in the WT PF-8–transfected and PF-8 ΔC–transfected cells; however, the activity of PARP1 in the PF-8 ΔN–transfected and PF-8 ΔI–transfected cells was similar to that in the control vector–transfected cells. In agreement with these results, the PARylation of RTA in the WT PF-8–transfected and PF-8 ΔC–transfected cells was significantly weaker when compared with the control vector–transfected, PF-8 ΔN–transfected, or PF-8 ΔI–transfected cells (Fig 4D). In contrast, PF-8 ΔN and PF-8 ΔI were capable of interacting with RTA, whereas PF-8 ΔC did not interact with RTA (Fig 4D). These findings suggest that the interaction between PF-8 and RTA may not be essential for PF-8–induced degradation of PARP1 and enhancement of the RTA activity. These results indicated that the dimerization domains (aa 1–27 and 277–304) of PF-8 are crucial to enhance RTA transactivation through the induction of PARP1 degradation, which attenuates PARP1 enzymatic activities.

### PF-8 interacts with cellular E3 ubiquitin ligases to target PARP1 for degradation

**Fig 5:**
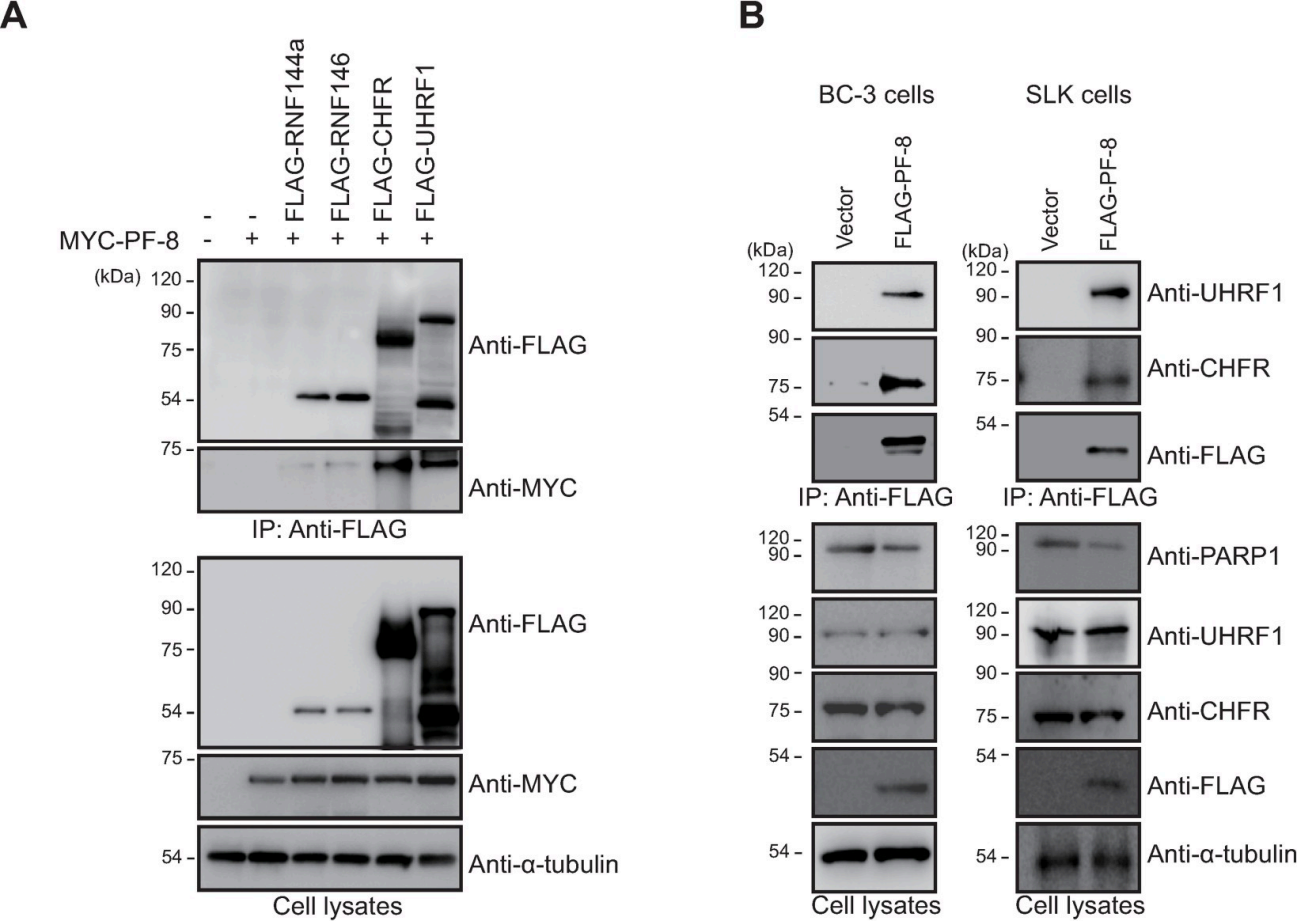
Identification of E3 ubiquitin ligases interacting with PF-8. (A) HEK293T cells were cotransfected with FLAG-tagged RNF144a, RNF146, CHFR, or UHRF1 and MYC-tagged PF-8. The transfected cells were harvested at 48 h post-transfection and assayed by coimmunoprecipitation (co-IP) with the anti-FLAG antibody. The cell lysates were analyzed by western blotting with the anti-FLAG-M2, anti-MYC, and anti-α-tubulin antibodies. (B) PF-8 interaction with endogenous CHFR and UHRF1. BC-3 cells or SLK cells were transduced with a FLAG-tagged PF-8 lentiviral vector. The cells were harvested and subjected to a co-IP assay with the anti-FLAG-M2 antibody. The cell lysates were analyzed by western blotting with the anti-FLAG-M2, anti-CHFR, anti-UHRF1, anti-PARP1, and anti-α-tubulin antibodies.

An analysis of PF-8 structure revealed that there is no known motif related to protein degradation, suggesting that PF-8 may recruit an additional cellular factor for PARP1 degradation [22]. We hypothesized that an E3 ubiquitin ligase is recruited by PF-8 to degrade PARP1. To test this hypothesis, we examined the cellular E3 ubiquitin ligases that are reported to interact with and ubiquitinate PARP1, e.g., checkpoint with FHA and RING finger domains (CHFR), ubiquitin-like with PHD and RING finger domains 1 (UHRF1), ring finger protein 144A (RNF144A), and RNF146 (also known as Iduna) [23–26] (S1 Fig). A co-IP analysis revealed that among these E3 ubiquitin ligases, CHFR and UHRF1 interacted with PF-8 (Fig 5A). PARP1 levels in cells cotransfected with PF-8 and either CHFR or UHRF1 were lower than those in cells transfected with PF-8, CHFR, or UHRF1 alone. The interactions between PF-8 and endogenous CHFR or UHRF1 were confirmed using cell lines BC-3 and SLK (endothelial-like cells) stably expressing FLAG-tagged PF-8 (Fig 5B and 5C). These data indicated that PF-8 interacts with PARP1-ubiquitinating E3 ligases: CHFR and UHRF1.

### CHFR is required for PF-8–induced PARP1 degradation and poly-ubiquitination

**Fig 6:**
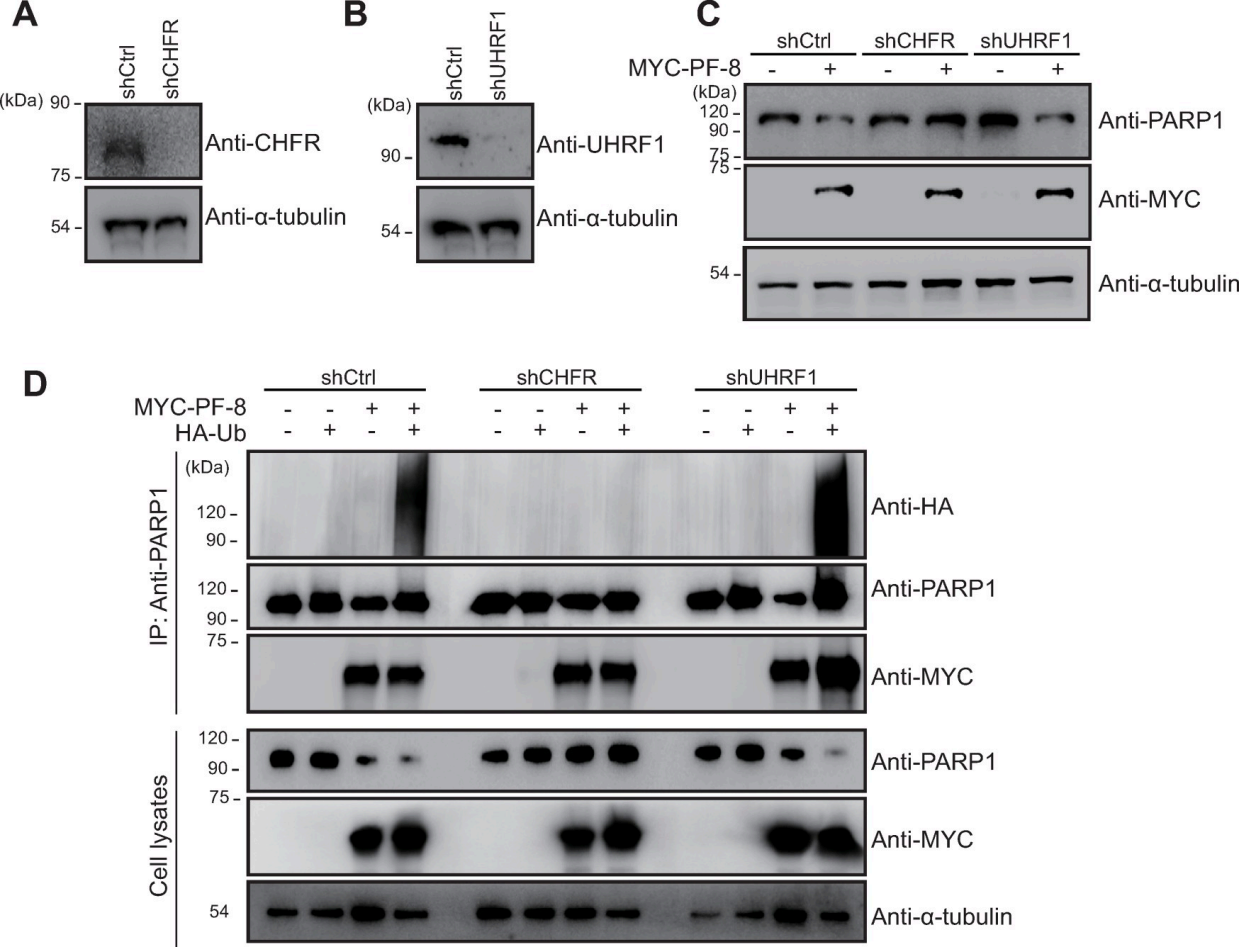
CHFR, but not UHRF1, is essential for PF-8–induced PARP1 poly-ubiquitination and degradation. (A and B) The construction of CHFR or UHRF1 knockdown cells. Knockdown HEK293T cells and control HEK293T cells were generated by transducing the cells with a lentiviral vector expressing shCHFR or shUHRF1 and a control short shRNA, respectively. The expression levels of CHFR (A) or UHRF1 (B) were analyzed by western blotting with anti-CHFR, anti-UHRF1, and anti-α-tubulin antibodies. (C) E3 ubiquitin ligase is essential for PF-8–mediated PARP1 degradation. The knockdown HEK293T cells were transfected with MYC-tagged PF-8. The transfected cells were harvested at 48 h post-transfection and subjected to western blotting with the anti-PARP1, anti-MYC, and anti-α-tubulin antibodies. (D) PF-8–mediated PARP1 poly-ubiquitination in the CHFR or UHRF1 knockdown cells. The knockdown HEK293T cells were transfected with MYC-tagged PF-8 and HA-tagged Ub. The transfected cells were harvested at 48 h post-transfection and assayed by immunoprecipitation with the anti-PARP1 antibody. The cell lysates were studied by western blotting with the anti-PARP1, anti-MYC, anti-HA, and anti-α-tubulin antibodies.

The role of these two E3 ubiquitin ligases in PF-8–induced PARP1 degradation was evaluated using either CHFR or UHRF1 knockdown HEK293T cells (shCHFR or shUHRF1) as well as control cells (shCtrl cells; Fig 6A and 6B). shCHFR cells did not exhibit PF-8–mediated PARP1 degradation, which was detectable in shCtrl cells, while PF-8–mediated PARP1 degradation levels in shUHRF1 cells were similar to those in shCtrl cells (Fig 6C). Consistent with these results, shCHFR cells showed attenuation of PF-8–induced poly-ubiquitination of PARP1; this effect was not observed in shUHRF1 cells (Fig 6D). It was also noted that PF-8 could bind to PARP1 even in the absence of CHFR or UHRF1 (Fig 6D). These results indicated that the E3 ubiquitin ligase CHFR takes part in PF-8–induced degradation and poly-ubiquitination of PARP1 through physical association.

**Fig 7:**
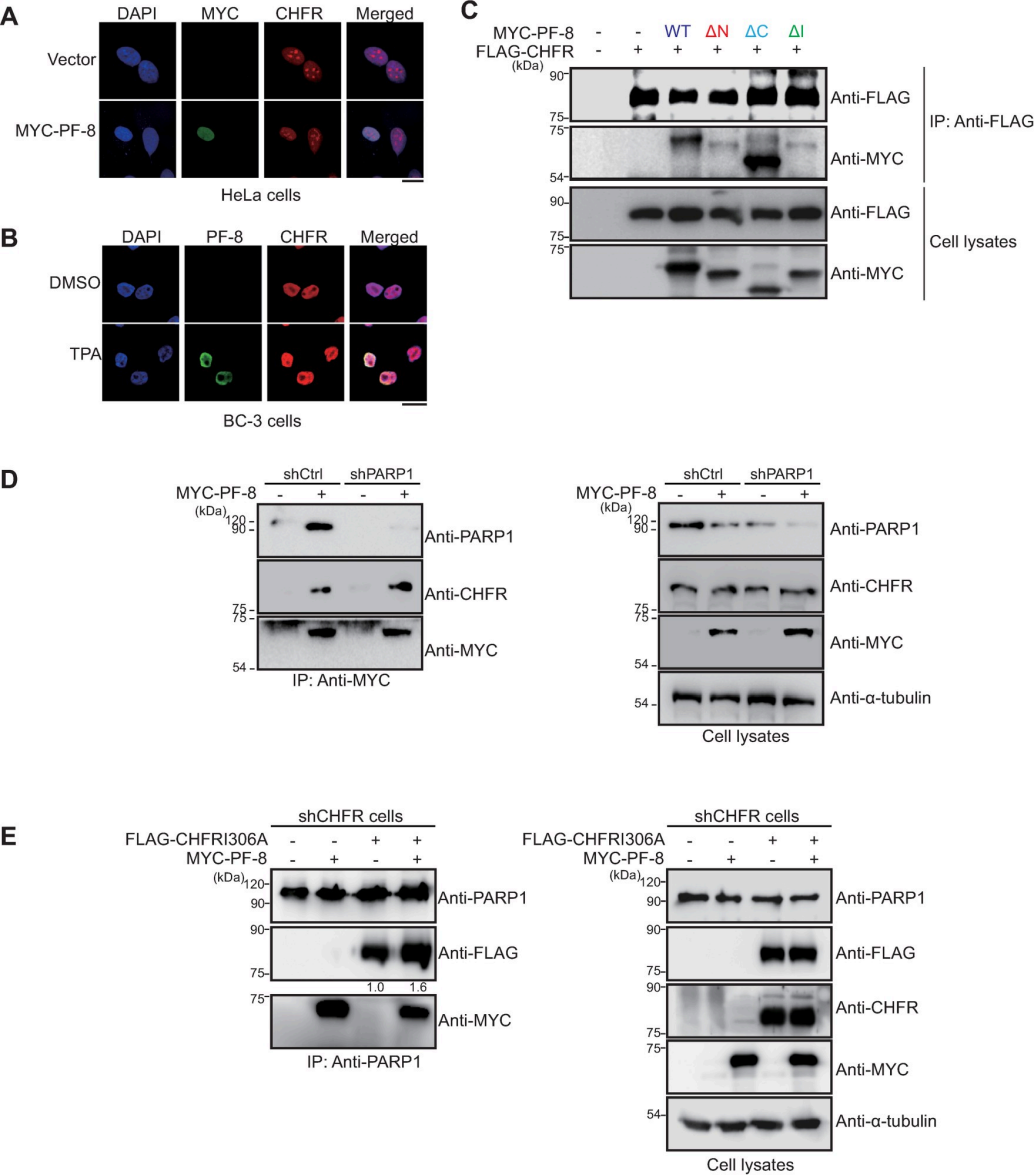
CHFR interacts with PF-8 independently of PARP1. (A) Subcellular localization of CHFR and PF-8. HeLa cells were transfected with the MYC-tagged PF-8, fixed at 48 h post-transfection, and immunostained with anti-MYC (green) and anti-CHFR (red) antibodies. The nuclei were stained with DAPI (blue). The samples were examined under the confocal laser scanning microscope. Scale bar, 20 μm. (B) Subcellular localization of CHFR and PF-8 in KSHV-replicating BC-3 cells. BC-3 cells were treated with TPA, fixed at 24 h post-treatment, and immunostained with anti-PF-8 (green) and anti-CHFR (red) antibodies. The nuclei were stained with DAPI (blue). The samples were examined under the confocal laser scanning microscope. Scale bar, 20 μm. (C) The interaction of CHFR with PF-8 mutants. HEK293T cells were transfected with MYC-PF-8 mutant and FLAG-CHFR constructs. The transfected cells were harvested at 48 h post-transfection and subjected to an immunoprecipitation (IP) assay with the anti-FLAG-M2 antibody. The cell lysates were analyzed by western blotting involving the anti-CHFR and anti-FLAG-M2 antibodies. (D) CHFR interaction with PF-8 in the shCtrl or shPARP1 cells. The PARP1 knockdown HEK293T (shPARP1) cells were transfected with MYC-tagged PF-8. The cells were harvested at 48 h post-transfection and assayed by IP using the anti-MYC antibody. The cell lysates were investigated by western blotting with the anti-CHFR, anti-PARP1, anti-MYC, and anti-α-tubulin antibodies. (E) Increased interaction of CHFR I306A and PARP1 by PF-8 in the shCHFR cells. The CHFR knockdown HEK293T (shCHFR) cells were transfected with FLAG-tagged CHFR I306A, a catalytic mutant of CHFR, and MYC-tagged PF-8. The transfected cells were harvested at 48 h post-transfection and assayed by co-IP using the anti-PARP1 antibody. Western blots were performed with the anti-PARP1, anti-FLAG, anti-CHFR, anti-MYC, and anti-α-tubulin antibodies. The relative levels of CHFR I306A in IP blots are presented in the absence and the presence of PF-8 in comparison with those in cell lysates blots.

Next, we characterized the interactions between PF-8 and CHFR. In line with the results of co-IP, MYC-tagged-PF-8–transfected HeLa cells showed nuclear colocalization of the PF-8 protein and endogenous CHFR (Fig 7A). Endogenous CHFR was co-localized with PF-8 in the nucleus of KSHV-replicating BC-3 cells (Fig 7B). The domains of PF-8 required for CHFR interactions in the transfected HEK293T cells were mapped using the PF-8 mutant constructs. The co-IP results revealed little or no interaction between CHFR and either PF-8 ΔN or PF-8 ΔI. Nonetheless, the interaction between PF-8 ΔC and CHFR was intact and was similar to that between PF-8 WT and CHFR (Fig 7C). The PF-8 domains required for the CHFR interaction turned out to be similar to those required for the PARP1 interaction. Hence, we hypothesized that PARP1 may be required for the interactions of PF-8 with CHFR. The shCtrl and shPARP1 cells were transfected with MYC-tagged PF-8. Results from co-IP assays revealed that PF-8 interacted with CHFR in both shCtrl and shPARP1 cells, suggesting that the PF-8–mediated CHFR recruitment was not dependent on PARP1 (Fig 7D). Next, we examined whether PF-8 recruits CHFR, which in turn increases the interaction between PARP1 and CHFR. Given that endogenous and transfected CHFR can target PARP1 for degradation, we conducted co-IP assays of PARP1 in shCHFR cells trans-complemented with CHFR I306A, a catalytic mutant of CHFR defective in E3 Ub-ligase activity [27]. Results showed that PF-8 increased the interaction between CHFR I306A and PARP1 (Fig 7E). These data suggested that CHFR is recruited by PF-8 to target PARP1 for protein degradation via direct physical interactions.

### CHFR is essential for PF-8–mediated enhancement of RTA transactivation activity

**Fig 8:**
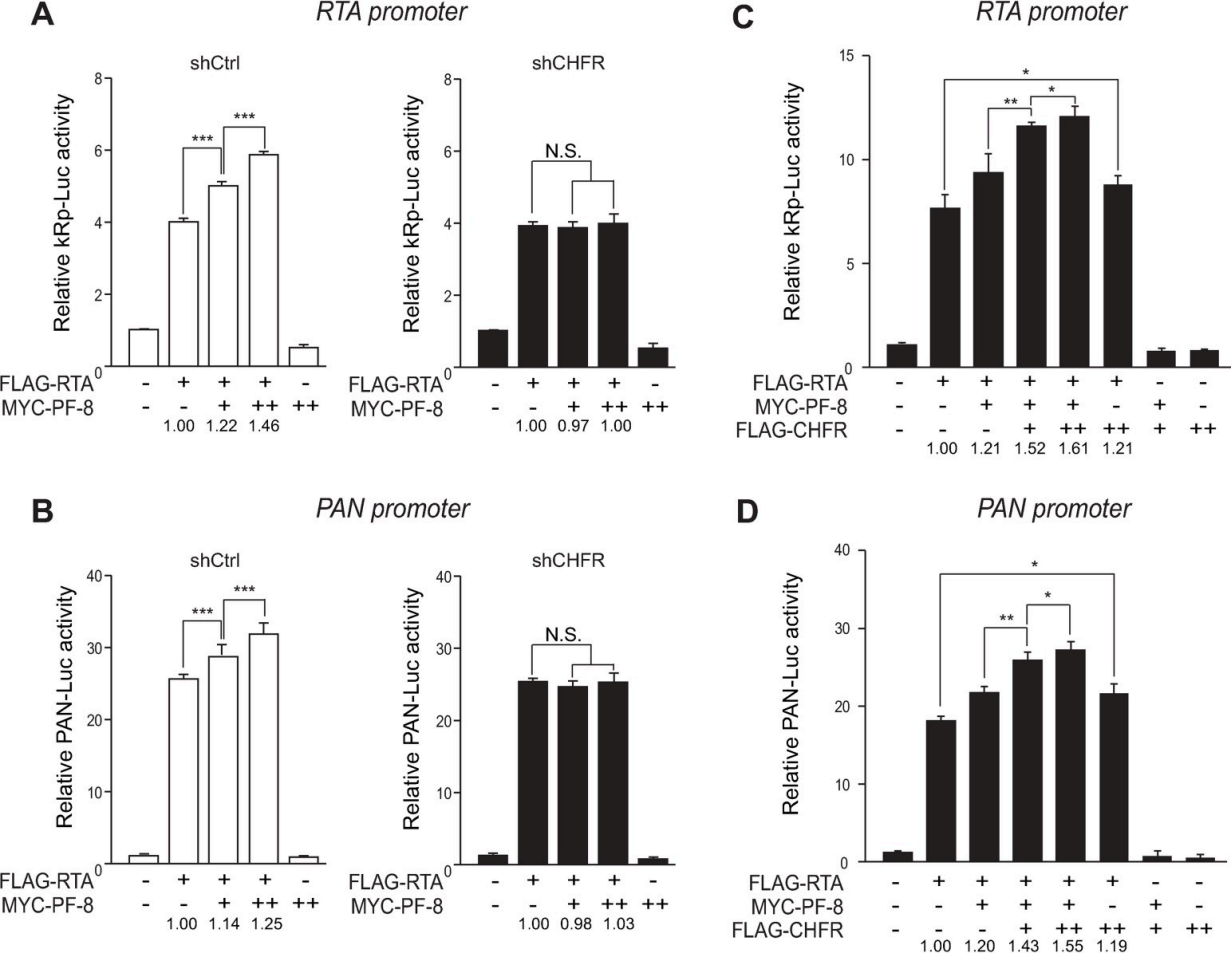
CHFR is required for PF-8–mediated enhancement of RTA transactivation activity. (A and B) Luciferase reporter assays with PF-8 in the shCHFR cells. The shCHFR or shCtrl HEK293T cells were cotransfected with reporter construct pGL3-kRP-Luc (A) or pGL3-PAN-Luc (B) (300 ng) and MYC-tagged PF-8 (150 or 300 ng) in the presence or absence of the FLAG-tagged RTA expression plasmid (25 ng). The cells were harvested at 48 h post-transfection for luciferase reporter assays. Each transfection was performed in triplicate, and the EGFP-expressing plasmid served as an internal control. The increased fold values of the promoter activity relative to the RTA alone are indicated. (C and D) Luciferase reporter assays with PF-8 and CHFR. HEK293T cells were cotransfected with reporter construct pGL3-kRP-Luc (C) or pGL3-PAN-Luc (D) (300 ng), MYC-tagged PF-8 (150 ng), and FLAG-tagged CHFR (10 or 20 ng) in the presence or absence of the FLAG-tagged RTA expression plasmid (25 ng). The cells were harvested at 48 h post-transfection for luciferase reporter assays. Each transfection was performed in triplicate, and the EGFP-expressing plasmid was used as an internal control. The increased fold values of the promoter activity relative to RTA alone are indicated. Statistical analysis was conducted by Student’s *t* test (*P < 0.05, **P < 0.01, and ***P < 0.005).

To further investigate the participation of CHFR in the PF-8–mediated enhancement of the RTA transactivation activity, reporter assays were conducted with *RTA* and *PAN* promoters. PF-8 did not enhance the RTA-mediated transactivation of both *RTA* and *PAN* promoters in shCHFR cells compared to that in control cells (Fig 8A and 8B). On the contrary, the PF-8–induced RTA-mediated transactivation in shUHRF1 cells was similar to that in shCtrl cells (S2A and S2B Fig). Moreover, the overexpression of CHFR dose-dependently increased RTA transactivation of *RTA* and *PAN* promoters in the presence of PF-8 (Fig 8C and 8D). In the absence of PF-8, the effect of CHFR on RTA transactivation was marginal, albeit statistically significant, suggesting that PF-8 recruitment of CHFR to PARP1 is critical for enhancement of RTA transactivation.

### CHFR is essential for efficient lytic replication of KSHV

**Fig 9:**
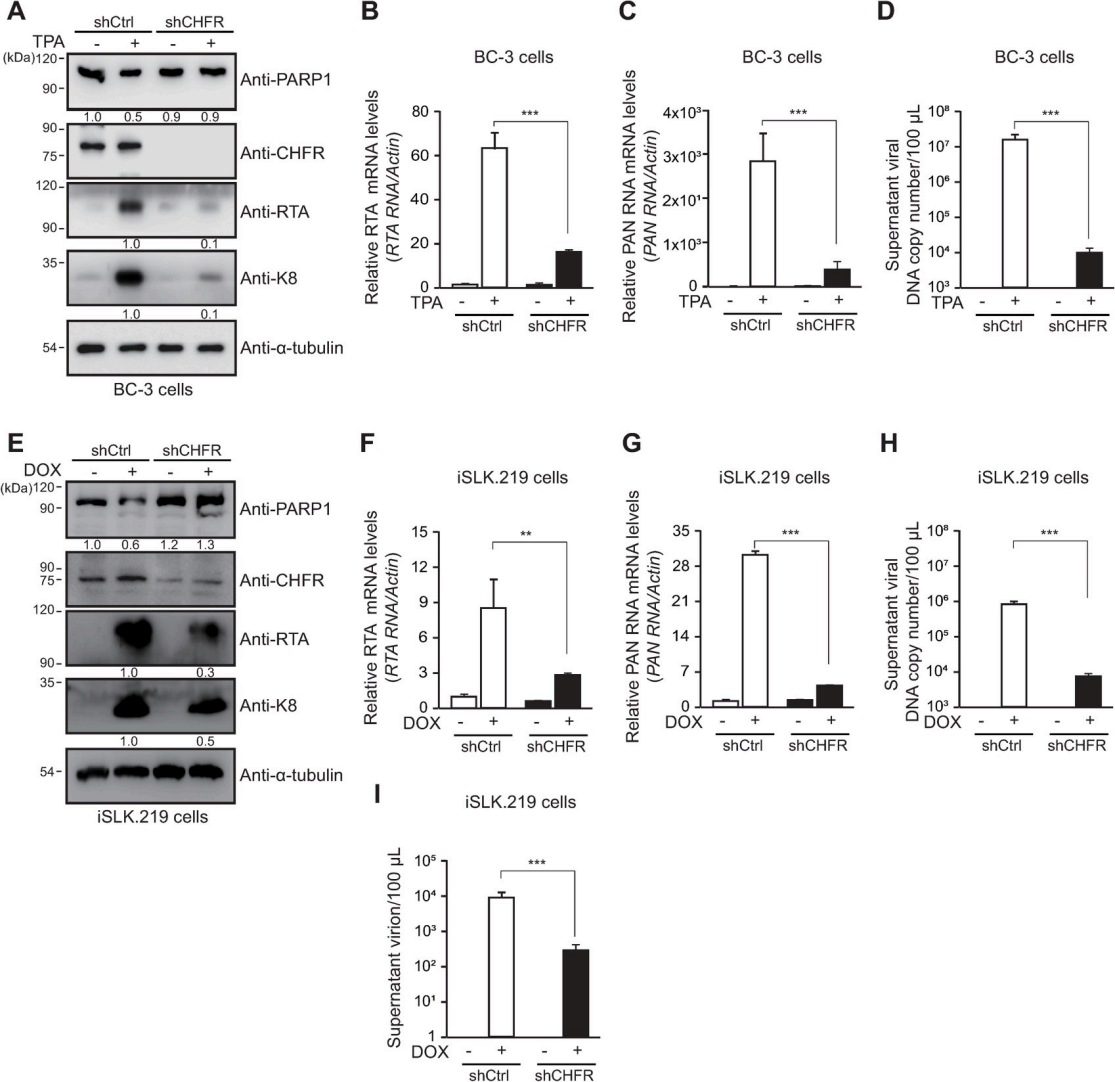
CHFR is essential for efficient KSHV lytic replication. (A to D) The CHFR knockdown BC-3 (shCHFR BC-3) cells and the control BC-3 (shCtrl BC-3) cells were generated by transduction with lentiviral vectors expressing the CHFR-targeting shRNA or control shRNA, respectively. At 24 h after TPA treatment, the lysates of shCtrl cells and shCHFR cells were processed for western blotting with the anti-PARP1, anti-CHFR, anti-RTA, anti-K8, and anti-α-tubulin antibodies (A). The protein levels of RTA or K8 relative to those of α-tubulin are indicated. The relative transcript levels of *RTA* (B) and *PAN RNA* (C) were analyzed by qRT-PCR. The viral DNA genome copy number in supernatants was quantified by qPCR using primers against PF-8 (D). (E to I) CHFR knockdown iSLK.219 (shCHFR iSLK.219) cells and control iSLK.219 (shCtrl iSLK.219) cells were generated as described for BC-3 cells. At 48 h after DOX treatment, the lysates of shCtrl iSLK.219 cells and shCHFR iSLK.219 cells were processed for western blotting as described in A (E). The relative transcript levels of *RTA* (F) and *PAN RNA* (G) were analyzed by qRT-PCR. Viral DNA genome copy number in supernatants harvested from iSLK.219 cells was quantified by qPCR using primers against PF-8 (H). The supernatants of the iSLK.219 cells were transferred to infect HEK293T cells. After 2 days, the number of GFP-positive HEK293T cells was analyzed using FACS to deduce titer of produced virion from induced iSLK.219 cells (I).

Next, we examined the role of CHFR on KSHV lytic replication in BC-3 cells. The KSHV reactivation did not induce PARP1 degradation in shCHFR BC-3 cells, but did so in shCtrl BC-3 cells generated (Fig 9A). The expressions of KSHV lytic proteins (RTA and K8) and transcripts (*RTA* and *PAN* RNA) were lower in shCHFR BC-3 cells than in shCtrl BC-3 cells following viral reactivation (Fig 9A–9C). The shCHFR BC-3 cells also produced the lower level of virion, as shown in viral genome copy from the culture supernatants (Fig 9D). The results from shCHFR iSLK.219 cells were consistent with those in shCHFR BC-3 cells (Fig 9E–9H). From shCHFR iSLK.219 cells, the culture supernatants were transferred to uninfected HEK293 cells and counted for the GFP (+) cells (Fig 9I). shCHFR iSLK.219 cells produced the lower amount of virion than shCtrl iSLK.219 cells. Taken together, these data indicate that CHFR is critical for efficient lytic replication of KSHV. Because CHFR was implicated in the PF-8–induced PARP1 degradation, we also examined the expression of CHFR after the viral reactivation. The CHFR level was not significantly affected by the KSHV reactivation in both iSLK.219 cells and BC-3 cells (S3 Fig).

These findings suggested that cellular CHFR recruited by viral PF-8 is essential for the PF-8–induced degradation of PARP1 and enhanced RTA transactivation activity, which promotes efficient lytic replication of KSHV.

## Discussion

In this study, we investigated the mechanism behind the attenuation (by a viral processivity factor, PF-8) of the negative regulatory effect of host PARP1 on KSHV lytic replication. A cellular E3 ubiquitin ligase (CHFR) that was recruited by PF-8 to facilitate lytic replication was identified. PARP1 is involved in diverse cellular pathways, including DNA damage response, cell death, proliferation, differentiation, gene transcription, and inflammation [5,14,28,29]. Additionally, PARP1 plays a key part in various viral infections, such as retrovirus, hepatitis B virus, and herpesvirus infections [28,30–37]. PARP1 exerts its inhibitory action on KSHV lytic replication by PARylating RTA and thereby inhibiting the transactivation activity of RTA [6,7,9,38,39]. PARP1, which is recruited to the terminal repeats of the KSHV genome, PARylates latency associated-nuclear antigen (LANA) to increase the latent viral genome copy number [8]. PARP1 can be a double-edged sword for the KSHV replication cycle; PARP1 positively regulates *oriLyt-*dependent DNA replication of KSHV, but suppresses the expression of genes partaking in the lytic replication cycle. Nonetheless, overall effects of these PARP1-mediated regulatory mechanisms decrease the production of infectious virions [9].

KSHV is believed to utilize two strategies to counter the inhibitory effect of PARP1 on lytic replication. In the first strategy, the *orf49-*encoded tegument protein, vPIP, sequesters PARP1 from RTA through direct interaction [40,41]. A similar strategy is used by the hepatitis B virus with the X protein [40–42]. Alternatively, the *orf59-*encoded viral processivity factor, PF-8, induces PARP1 degradation and promotes RTA-mediated transactivation [10]. Compared to other viral factors that are known to modulate the PARP1 activity via direct association [40–42], PF-8 is unique in that it decreases the PARP1 activity by degrading PARP1. The viral processivity factor PF-8 usually assists viral DNA polymerase during genome replication. On the contrary, the role and mechanism of action of PF-8, which has no known motif for engagement of the UPS to induce protein degradation, have not been elucidated until our study.

Given that PARP1 acts as an important component of DNA damage response, PF-8-induced PARP1 degradation may be an indirect effect through DNA damage response pathways. We checked the DNA damage response in HEK293T cells expressing PF-8 by observing phosphorylation of H2AX (γH2AX) and 53BP1 recruitment [43]. PF-8 alone neither induced phosphorylation of H2AX nor changed 53BP1 localization (S4A and S4B Fig). In addition, treatment with an ATM inhibitor (KU55933) did not affect PF-8-mediated PARP1 degradation and interaction between PF-8 and PARP1 (S4C Fig). These results suggest that PF-8–induced PARP1 degradation via physical association is unlikely due to an indirect effect of PARP1 through activation of DNA damage response pathways. In contrast, PF-8–mediated PARP1 degradation may affect the recruitment of DNA damage repair machinery during lytic replication. Hollingworth *et al*. reported that nonhomologous end joining (NHEJ) repair proteins such as Ku80 and DNA-PK restricted KSHV lytic replication [44]. PARP1 recruits Ku80 on double-strand breaks (DSB) and facilitates DSB repair and PF-8 blocks interaction between Ku70/80 and DNA-PKcs [45], thereby inhibiting NHEJ, it is plausible to think that PF-8–mediated PARP1 degradation may ameliorate the suppressive effect of NHEJ components, Ku80 and DNA-PKcs on virus replication.

Various studies suggest that viruses employ the host UPS to overcome host barriers to viral infection [46,47]. KSHV utilizes viral proteins as E3 ubiquitin ligases to recruit some components of the UPS to promote own replication [48–51]. In lytic replication, E3 ligase proteins K3 and K5, members of the membrane-associated RING-CH (MARCH) family, reduce the expression of cell surface molecules, including MHC-I, ICAM-I, B7.2, CD83, and CD4, to evade host immune surveillance [48–51]. RTA functions as an E3 ubiquitin ligase and causes degradation of cellular repressors, such as K-RBP, Hey1, and interferon-regulatory factor 7 (IRF7), to promote lytic replication [52–54]. During latency, KSHV-encoded LANA recruits the EC5S ubiquitin complex to degrade tumor suppressor proteins, such as p53 and von Hippel-Lindau (VHL), and thus may facilitate tumorigenesis [55]. Recently, LANA was also reported to physically associate with a cellular E3 ligase, RLIM (RING finger LIM-domain-interacting protein), and to induce its autoubiquitination for its degradation [56]. Although a liquid chromatography–mass spectrometry study [57] and our unpublished proteomic studies on the PF-8 interactome have not revealed any interacting E3 ligases, our current findings indicate that a viral factor engages the host UPS to degrade a cellular protein. Among PARP1 poly-ubiquitinating E3 ligases, CHFR and UHRF1 were found to interact with PF-8 (Fig 4). Our IFA results for transfected MYC-PF-8 and virus-encoded PF-8 in BC-3 cells showed co-localization of PARP1, CHFR and PF-8. If possible, an assay for *in situ* detection of endogenous protein interaction like proximity ligation assay would give more supportive results to validate their interaction. Although IFA showed PF-8 from KSHV replicating cells, we could not detect PF-8 protein expressed from KSHV in Western blot analysis even with the same PF-8 antibody used in IFA. Due to this limitation, we were only able to show interaction of endogenous PARP1 or CHFR with MYC- or FLAG-tagged PF-8 in Western blot. Our gene knockdown experiments showed that an E3 ubiquitin ligase CHFR is essential for PF-8–mediated PARP1 degradation, which promotes efficient lytic replication (Fig 5). IP-assays with CHFR I306A in shCHFR cells and reporter assays suggest that PF-8 recruits CHFR to enhance the interaction with PARP1, which results in promotion of PARP1 degradation and RTA transactivation. Although UHRF1 is dispensable for the PF-8–induced PARP1 degradation, the interaction between PF-8 and UHRF1 may also modulate the PARP1 activity through an unknown mechanism and may play additional roles in viral replication. CHFR is a RING-type E3 ubiquitin ligase that acts as a mitotic-checkpoint factor and a tumor suppressor [27,58–61]. Nevertheless, the function of CHFR in viral replication or pathogenicity has not been documented before our study. To the best of our knowledge, this is the first study to report that the E3 ubiquitin ligase CHFR is utilized by the virus to promote its replication.

**Fig 10:**
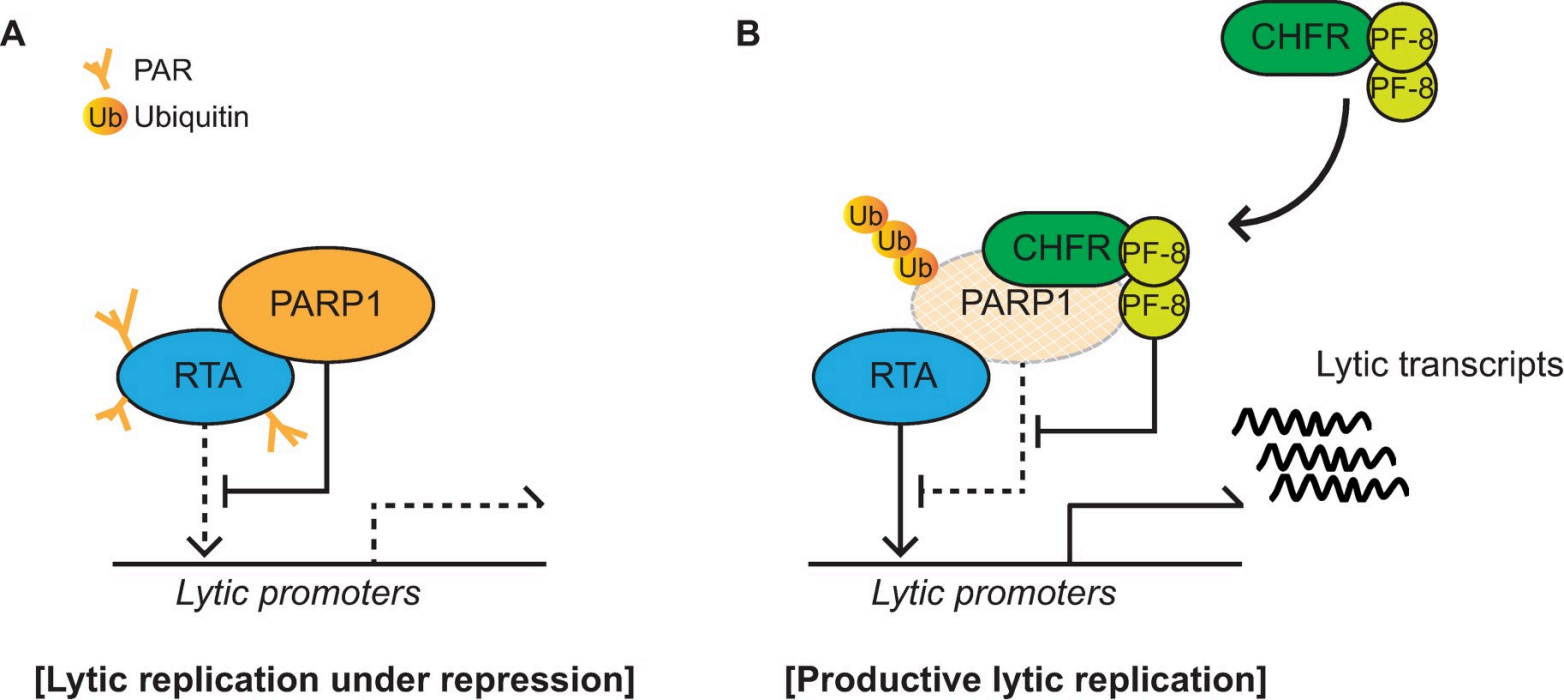
A schematic diagram depicting PF-8-induced PARP1 degradation via recruitment of CHFR to promote KSHV lytic replication. (A) PARP1 interacts with RTA and inhibits RTA function through PARylation of RTA. Lytic replication is under repression. (B) PF-8 is expressed as an early gene product of KSHV lytic genes. PF-8 interacts with CHFR, an E3 ligase, and recruits it to target PARP1, and resulted in polyubiquitination and degradation of PARP1. PF-8-induced PARP1 degradation derepresses the RTA activity and promotes efficient lytic replication of KSHV.

The pull-down assays of PARP1 mutant constructs revealed that the interaction between PARP1 and PF-8 is mediated by the AD (Fig 2). The BRCT motif of PARP1 in the AD is a conserved motif among many other protein motifs participating in the cell cycle and DNA damage response [62]. Additionally, the BRCT motif of PARP1 is known to mediate protein–protein interactions among DNA repair proteins, such as XRCC1 and DNA ligase III-α [63–65]. Our study proved that PF-8, a viral processivity factor, binds to PARP1 through association with the BRCT motif. Our experiments on PF-8 deletion mutants show that aa 1–27 and 277–304, which are missing in mutants PF-8 ΔN and PF-8 ΔI, respectively, are critical for PARP1 degradation and association between PF-8 and PARP1 (Fig 3). Additionally, the interaction of PF-8 with PARP1, but not with RTA, turned out to be crucial for PF-8–mediated upregulation of lytic genes. Crystal structure of PF-8 has revealed that these domains contain a β-sheet (βA_1_, aa 7 to 11) and an α-helix (αA_1_, aa13 to 30) at the N terminus and two β-sheets (βH_2_, aa 278 to 283; βI_2_, aa 291 to 297) in an internal region [22]. Thus, the functions of mutant proteins PF-8 ΔN and PF-8 ΔI may be defective due to the disruption of overall protein folding. On the contrary, the results of this study indicate that mutant proteins PF-8 ΔN and PF-8 ΔI are deficient in PARP1 interaction and degradation but not in terms of protein expression, nuclear localization, and interaction with RTA. These data imply that these mutant proteins may hold proper protein folding sufficient enough to maintain certain biological activities (Fig 3). In contrast, the PF-8 ΔC mutant protein was found to be as functional as the WT in terms of PARP1 degradation induction and the reduction in the level of PARylated RTA but deficient in the interaction with RTA, thereby helping us to further narrow down the RTA interaction domain of PF-8 from aa 266–396 to aa 369–396 [18]. Interestingly, the same domain of PF-8 associated with PARP1 is also required for CHFR interaction. Given that PF-8 dimer presents two identical interaction domains away from the dimeric interface, based on the crystal structure [22], PF-8 dimer may interact with PARP1 and CHFR via the same domain of each monomer regions, facilitating interaction between PARP1 and CHFR. Our proposed working model is depicted in Fig 10.

In conclusion, this study shows that KSHV PF-8 recruits host CHFR (a cellular E3 ubiquitin ligase) to target PARP1 for proteasomal degradation, thereby promoting efficient KSHV lytic replication. The domain deletion experiments proved that the same domains of PF-8 which directly associate with PARP1 and CHFR, are crucial for the induction of PARP1 degradation (Figs 3 and 7). Therefore, this study revealed a novel mechanism via which a viral processivity factor facilitates lytic replication as well as the importance of PARP1 and CHFR for the regulation of the gammaherpesvirus replication cycle.

## Materials and methods

### Cell culture

Cell lines HEK293T (human embryonic kidney cells), HeLa (human epithelial cells), SLK (endothelial-like cells), and iSLK.219 (KSHV-positive SLK cells) were cultured in complete Dulbecco’s modified Eagle’s medium (DMEM) (PAN) supplemented with 10% of fetal bovine serum (FBS; HyClone) and penicillin and streptomycin (10 U/mL; HyClone). The SLK and iSLK.219 cells are a kind gift from Dr. Jinjong Myoung (Korea Zoonosis Research Institute, Chonbuk National University, Republic of Korea) [11]. BC-3 cells (KSHV-positive B cells) were cultured in the complete RPMI 1640 medium (Welgene) supplemented with 10% FBS (Atlas) and penicillin and streptomycin (10 U/mL; HyClone).

### Plasmids and cloning

To generate WT PF-8 and mutant constructs PF-8 ΔN and PF-8 ΔC, genomic DNA from BC-3 cells was subjected to polymerase chain reaction (PCR) amplification with the primers listed in Table 1, and each PCR product was cloned into the pENTR-3C vector. The PF-8 ΔI DNA construct was cloned by a two-step PCR method. In the first PCR, DNA amplification was performed with the following primer pairs: PF-8 WT-F and PF-8 ΔI-R as well as PF-8 ΔI-F and PF-8 WT-R. These two amplicons were subjected to a second PCR, with the PF-8 WT F and R primer pair. The resultant amplicon was a DNA fragment with the deletion of a sequence encoding aa 277–304 of PF-8 (PF-8 ΔI mutant). To generate the destination vector harboring FLAG-tagged or MYC-tagged PF-8 or its mutants, the PCR-amplified DNA fragments were cloned into the pENTR3C plasmid. The clones were next transferred to a FLAG-tagging (pTAG-attRC1) or a 6 MYC-tagging (pCS3-MT-6-MYC) destination vector to generate the FLAG-tagged or MYC-tagged PF-8 constructs by the Gateway technology (Invitrogen, Carlsbad, CA, USA), following the manufacturer’s instructions. To construct the pCMV2-FLAG clones, the amplicons were cloned between the *Hin*dIII and *Eco*RI sites of the pCMV2-FLAG vector. The C274-FLAG-PF8 construct was prepared for lentiviral transduction. The FLAG-tagged-PF-8–encoding DNA fragment, which was PCR-amplified from the pCMV2-FLAG-PF8 plasmid, was cloned into the C274 plasmid by means of the primers listed in Table 1. RNF144A and RNF146 expression constructs were cloned into the pENTR3C vector using the cDNA of BC-3 cells, which was subjected to PCR with the following primers: RNF144A-F (5′-ATTAGGATCCATGACCACAACAAGGTAC-3′) and RNF144A-R (5′-ATAACTCGAGCTAGGTGGGTAACGGG-3′) for RNF144A [66] and RNF146-F (5′-GGCTACTGAATTCATGGCTGGCTGTGGTG-3′) and RNF146-R (5′-CCATCAGCGGCCGCTTAAACTTCAGTTACTG-3′) for RNF146 (GenBank accession number: CR533514.1). These clones were then transferred to a FLAG-tagging (pDEST-SG5-FLAG) destination vector via the Gateway technology. For the CHFR knockdown, the shCHFR construct (expressing short hairpin RNA; shRNA) was generated using the following primer pair: shCHFR-F (5′-CCGGAGCATAAGTTTACAGCCTACACTCGAGTGTAGGCTGTAAACTTATGCTTTTTTG-3′) and shCHFR-R (5′-AATTCAAAAAAGCATAAGTTTACAGCCTACACTCGAGTGTAGGCTGTAAACTTATGCT-3′). The construct was then inserted into the pLKO.1 TRC cloning vector, following the TRC cloning protocol [59]. The sequences of the clones were verified by conventional sequencing.

**Table 1:** Primers used for PF-8 cloning.

Cloning vector	Primer name	Primer Sequence
pENTR-3C	PF-8 WT-F	5′-CCGGAATTCATGCCTGTGGATTTTCACTATGGGG-3′
	PF-8 WT-R	5′-GGGGCGGCCGCTCAAATCAGGGGGTTAAATG-3′
	PF-8 ΔN-F	5′-CCGATGAGAATTCAGTGCCACTAAAACCGG-3′
	PF-8 ΔC-R	5′-CAATTGCGGCCGCTTATGTGCTGTCCTTAGTTGG-3′
	PF-8 ΔI-F	5′-CCAACGAAATATCTGCCGAGGGAGGCGAGTCTTCG-3′
	PF-8 ΔI-R	5′-CCCTCGGCAGATATTTCGTTGGAGTGCCAAATC-3′
pCMV2-FLAG	PF-8 FLAG-F	5′-CCCAAGCTTGCAGCTGCAATGCCTGTGGATTTTCAC-3′
	PF-8 FLAG-R	5′-GGCTACTGAATTCTCAAATCAGGGGGTTAAATGT-3′
	PF-8 ΔN FLAG-F	5′-CCCAAGCTTGCAGCTGCAATGAGTGCCACTAAAACC-3′
	PF-8 ΔC FLAG-R	5′-GGCTACTGAATTCTCATGTGCTGTCCTTAGTTGG-3′
C274	C274-FLAG PF-8 F	5′-GCCAATATAGCTAGCACCATGGACTACAAAGACG-3′
	C274 PF-8 R	5′-GGATACTGCGGCCGCAATCAGGGGGTTAAATGT-3′

Hemagglutinin (HA)-tagged ubiquitin and its mutant constructs were provided by Dr. Jin-Hyun Ahn (Sungkyunkwan University, Republic of Korea). The pCMV5-FLAG-PARP1 domain mutant constructs were obtained from Dr. Mi-Ock Lee (Seoul National University, Republic of Korea) [40–42]. The pCMV5-FLAG-PARP1ΔAD construct was generated by a two-step PCR approach. PARP1 DBD sequence was amplified by primers: F-5’-GAAAGATCTGATGGC GGAGTCTTCGGATA-3’ and R-5’-GCTCCTCCTTTAAGAGTTAAGGAGGGCGGAGGCGTGGCCG-3’. PARP1 CAT sequence was amplified by primers: F-5’-CGGCCACGCCTCCGCCCTCCTTAACTCTTA AAGGAGGAGC -3’ and R-5’- GAATCTAGATTACCACAGGGAGGTCTTAA -3’. In the next PCR step, two PCR products were mixed as templates and amplified by primers: F-5’- GAAAGATCTGATGGCGGAGTCTTCGGATA-3’ and R-5’-GAATCTAGATTACCACAGGGAGGTCTTAA-3’. The amplicons were cloned between the *Bgl*II and *Xba*I sites of the pCMV5-FLAG vector. The FLAG-tagged CHFR and CHFR I306A constructs were provided by Dr. Jae Hong Seol (Seoul National University, Republic of Korea) [67]. The FLAG-tagged UHRF1 and shUHRF1 constructs were provided by Dr. Sang-Beom Seo (Chung-Ang University, Republic of Korea) [68]. The shPARP1 knockdown construct and control construct were obtained from Dr. Lee Kraus (the University of Texas Southwestern Medical Center, Dallas, TX, USA).

### Transfection and transduction

HEK293T cells were transfected using polyethylenimine (1 mg/mL) (PEI; Polysciences, Inc, Warrington, PA, USA). Briefly, DNA was incubated with PEI in the ratio of 1:5 for 20 min at room temperature in the DMEM. The HEK293T cells were treated with this mixture. HeLa cells were transfected by means of the PEI complex at pH 4.0 [69]. To construct HA-Ub-expressing BC-3 cells, the cells were transfected with via electroporation. Electroporation was performed at 1,350 V for 40 ms using a Microporator MP-100 (Digital Bio) according to the manufacturer’s instructions. The transfected cells were incubated for the various time and subjected to further assays. To produce the lentiviruses for transduction, HEK293T cells were cotransfected with the lentiviral construct (C274-FLAG-PF-8, 53BP1trunc-Apple or various shRNA-encoding plasmids) and packaging plasmids psPAX2 and pMD2.G [43,70]. The culture medium containing the lentivirus was centrifuged at 500 *g* for 5 min three times every 24 h. The supernatant was passed through a 0.45 μm capsule filter (Sartorius). The supernatant was then incubated with target cells to express the FLAG-tagged PF-8, 53BP1trunc-Apple or to knock down a target gene. The transduced cells were selected in the medium containing 1 μg/mL puromycin. The C274-FLAG-PF-8 transduced cells were sorted based on green-fluorescence signal using sorted by a FACSAria (BD Bioscience). iSLK.219 cells were transduced with the shCHFR or shCtrl lentiviral vector without puromycin selection [10].

### Luciferase reporter assays

A Luciferase Reporter Assay System (Promega, Madison, WI, USA) was used to measure the activities of the KSHV *PAN* promoter and the *RTA* promoter [20,21]. HEK293T cells were cotransfected with a promoter–luciferase construct and RTA and/or PF-8 expression constructs. The cells were harvested at 48 h post-transfection and analyzed for luciferase activity, following the manufacturer’s instructions (Promega). Each transfection was performed in triplicate, and EGFP served as an internal control.

### Quantitative reverse-transcription PCR (qRT-PCR)

Total RNA was extracted from the cells using the TRI reagent (Molecular Research center, Cincinnati, OH, USA), in accordance with the manufacturer’s instructions. cDNAs were synthesized from the isolated RNA with the RevertAid First Strand cDNA Synthesis Kit (Invitrogen) and random hexamers. The transcripts were quantified via a Rotor-Gene qRT-PCR Detection System (Qiagen, Hilden, Germany). The qRT-PCR analysis was performed with the following primer pairs: *PF-8* (Forward, 5′-CTCCCTCGGCAGACACAGAT-3′; Reverse, 5′-GCGTGGTGCACACCGACGCCC-3′), *RTA* (Forward, 5′-GTGGCAATGAGGATGACTTGTTC-3′; Reverse, 5′-TAGTGGTGGTCGGAGATTCGTA-3′) and *PAN RNA* (Forward, 5′- ATAGGCGACAAAGTG AGGTGGCAT-3′; Reverse, 5′- TAACATTGAAAGAGCGCTCCCAGC-3′) [71]. The expression level of a transcript was normalized to that of *ACTB (β-actin)* mRNA, which was amplified with the following primer pair: Forward, 5′-GTATCCTGACCCTGAAGTACC-3′; Reverse, 5′-TGAAGGTCTCAAACATGATCT-3′. The qRT-PCR was carried out using SYBR green. The thermal cycling conditions were as follows: 95°C for 15 min, followed by 50 cycles of 95°C for 10 s, 55°C for 15 s, and 72°C for 20 s. The qRT-PCR analysis was followed by a melting curve analysis, following the manufacturer’s instructions.

### Quantitative real-time PCR

Total DNA was isolated from KSHV-replicating cell culture media using QIAamp DNA Mini kit (Qiagen). *ORF59* locus specific primers (Forward, 5′-CTCCCTCGGCAGACACAGAT-3′; Reverse, 5′-GCGTGGTGCACACCGACGCCC-3′) were used to determine the copy numbers of viral genomic DNAs. The real-time PCR was carried out using SYBR green. The thermal cycling conditions were as follows: 95°C for 15 min, followed by 50 cycles of 95°C for 10 s, 55°C for 15 s, and 72°C for 20 s. The viral DNA copy number was calculated via a Rotor-Gene qRT-PCR Detection System (Qiagen, Hilden, Germany).

### Western blotting analysis

Whole-cell lysates were subjected to sodium dodecyl sulfate polyacrylamide gel electrophoresis. The resolved proteins were transferred to a polyvinylidene fluoride membrane (0.45 μm pore size). The membrane was probed with primary antibodies: anti-FLAG-M2 (1:2,000; Sigma, St. Louis, MO, USA), anti-MYC (1:500; laboratory-made or 1:2,000; Roche), anti-RTA (1:500; laboratory-made), anti-K8 (1:500; laboratory-made), anti-GFP (1:500; Santa Cruz Biotechnology, Dallas, TX, USA), anti-PARP1 (1:1,000; BD Biosciences), anti-PAR (1:500; Trevigen), anti-CHFR (1:1,000; Cell Signaling Technology, Danvers, MA, USA), anti-UHRF1 (1:500; Santa Cruz Biotechnology), anti-H2AX (1:500; Cell Signaling Technology, Danvers, MA, USA), anti-γH2AX (1:500; Merck Millipore, Billerica, MA, USA) or anti-α-tubulin (1:2,000; Sigma). The membrane was then incubated with the horseradish peroxidase–conjugated goat anti-rabbit or goat anti-mouse immunoglobulin G antibody (1:5000; a secondary antibody; Santa Cruz Biotechnology). The protein bands were detected with enhanced chemiluminescence (ECL) and western blotting detection reagents (ELPIS, Taejeon, Republic of Korea). The protein bands were documented on an LAS-4000 chemiluminescent image analyzer (Fujifilm). The band intensities were calculated in the ImageJ software [72].

### The co-IP assays

The transfected or transduced cells were incubated at 4°C for 1 h with IP lysis buffer (20 mM HEPES-KCl pH 7.4, 100 mM NaCl, 0.5% of Nonidet P-40, and 1% of Triton X-100) supplemented with a protease inhibitor cocktail (1:100; Sigma). The cell lysates were centrifuged at 12,000 *g* and 4°C for 10 min. The supernatant was incubated with the various antibodies at 4°C for 1 h in a shaker. Next, the samples were mixed with protein A/G agarose beads (Santa Cruz Biotechnology) and kept at 4°C for 16 h. After that, the beads were washed with IP buffer, and the proteins were analyzed by western blotting.

### The immunofluorescence assay and confocal microscopy

HeLa cells were seeded onto a cover glass in a 24-well plate for 24 h before transfection. The DNA constructs were transfected into the cells by the PEI transfection method for 48 h. The cells were fixed for 15 min with 4% paraformaldehyde and 0.15% picric acid in phosphate-buffered saline (PBS) and blocked with 10% normal goat serum prepared in PBS containing 0.3% of Triton X-100 and 0.1% of bovine serum albumin. The TPA or DMSO treated BC-3 cells were harvested, washed with PBS, and fixed for 10 min in cold acetone. The fixed cells were washed again with PBS and air-dried. Then the cells were blocked with 10% normal goat serum prepared in PBS containing 0.3% of Triton X-100 and 0.1% of bovine serum albumin. Next, the cells were incubated with the anti-MYC (1:200), anti-PF-8 (1:100; a kind gift from Dr. Bala Chandran at University of South Florida (Tampa, Florida, USA)), anti-CHFR (1:100), and anti-PARP1 (1:800; Cell Signaling Technology) antibodies for 16 h at 4°C, followed by probing with secondary antibodies (anti-mouse-Cy3 and anti-rabbit-Rho; 1:2,000; Jackson Immuno Research, West Grove, PA, USA) for 45 min at room temperature. After that, the cells were incubated with 4′,6-diamino-2-phenylindole (DAPI; 1:1000) for nuclear staining. Fluorescence images were captured at a magnification of 1000× under a confocal laser scanning microscope (LSM 5 Exciter, Zeiss).

## Supporting information

S1 Fig E3 ubiquitin ligases interact with PARP1. PARP1 interaction with cellular E3 ubiquitin ligases. HEK293T cells were transfected with FLAG-tagged RNF144a, RNF146, CHFR, or UHRF1. The transfected cells were harvested at 48 h post-transfection and subjected to an immunoprecipitation assay with the anti-FLAG antibody. The cell lysates were analyzed by western blotting with the anti-FLAG-M2, anti-PARP1, and anti-α-tubulin antibodies.
(TIF)

S2 Fig UHRF1 is dispensable for PF-8–mediated enhancement of replication and transcription activator (RTA) transactivation activity. (A and B) Luciferase reporter assays of PF-8 in shUHRF1-transfected cells. The shUHRF1-transfected or shCtrl-transfected HEK293T cells were cotransfected with reporter construct pGL3-kRP-Luc (A) or pGL3-PAN-Luc (B) (300 ng) and MYC-tagged PF-8 (150 or 300 ng) in the presence or absence of the FLAG-tagged RTA expression plasmid (25 ng). The cells were harvested at 48 h post-transfection for luciferase reporter assays. Each transfection was performed in triplicate, and the EGFP-expressing plasmid served as an internal control. Statistical analysis was carried out by Student’s *t* test (*P < 0.05, **P < 0.01, and ***P < 0.005).
(TIF)

S3 Fig CHFR expression upon Kaposi’s sarcoma–associated herpesvirus (KSHV) reactivation. iSLK.219 cells and BC-3 cells latently infected with KSHV were treated with doxycycline (DOX) for 48 h or 12-O-tetradecanoylphorbol-13-acetate (TPA) for 24 h to induce viral reactivation. The cells were harvested and assayed by western blotting with the anti-PARP1, anti-CHFR, anti-RTA, anti-K8, and anti-α-tubulin antibodies.
(TIF)

S4 Fig PF-8 does not induce DNA damage response. (A) Phosphorylation of H2AX in SLK cells. SLK cells were transduced with a FLAG-tagged PF-8 or control lentiviral vector. As a control, 1 mM H_2_O_2_ was treated for 30 min. The cells were harvested and analyzed by western blotting with the anti-γH2AX, H2AX anti-FLAG-M2 and anti-α-tubulin antibodies. (B) 53BP1 recruitment in HEK293T cells. DNA damage reporter HEK293T cells were generated by transducing the cells with a lentiviral vector expressing truncated 53BP1 (amino acids 1220–1711) to Apple fluorescent protein. The cells were transfected with FLAG-tagged PF-8 or treated with 1 mM H_2_O_2_ for 30 min. The samples were examined for red-fluorescence under a fluorescence microscope (Leica DM IL LED fluo, Leica). Scale bar, 20 μm. (C) PARP1 degradation and interaction with PF-8 upon ATM kinase inhibitor treatment. HEK293T cells were transfected with MYC-tagged PF-8. After 32 h post-transfection, media were changed and the cells were treated with 10 μM KU55933 for 16 h. The cells were harvested and assayed by IP using the anti-PARP1 antibody. The cell lysates were analyzed by western blotting with the anti-PARP1, anti-MYC, and anti-α-tubulin antibodies.
(TIF)
