# Supplementary figures and images for: Kaposi’s sarcoma-associated herpesvirus processivity factor (PF-8) recruits cellular E3 ubiquitin ligase CHFR to promote PARP1 degradation and lytic replication
Source: PLoS Pathog. 2021 Jan 28;17(1):e1009261. doi: 10.1371/journal.ppat.1009261 (PMC7872283; doi:10.1371/journal.ppat.1009261)

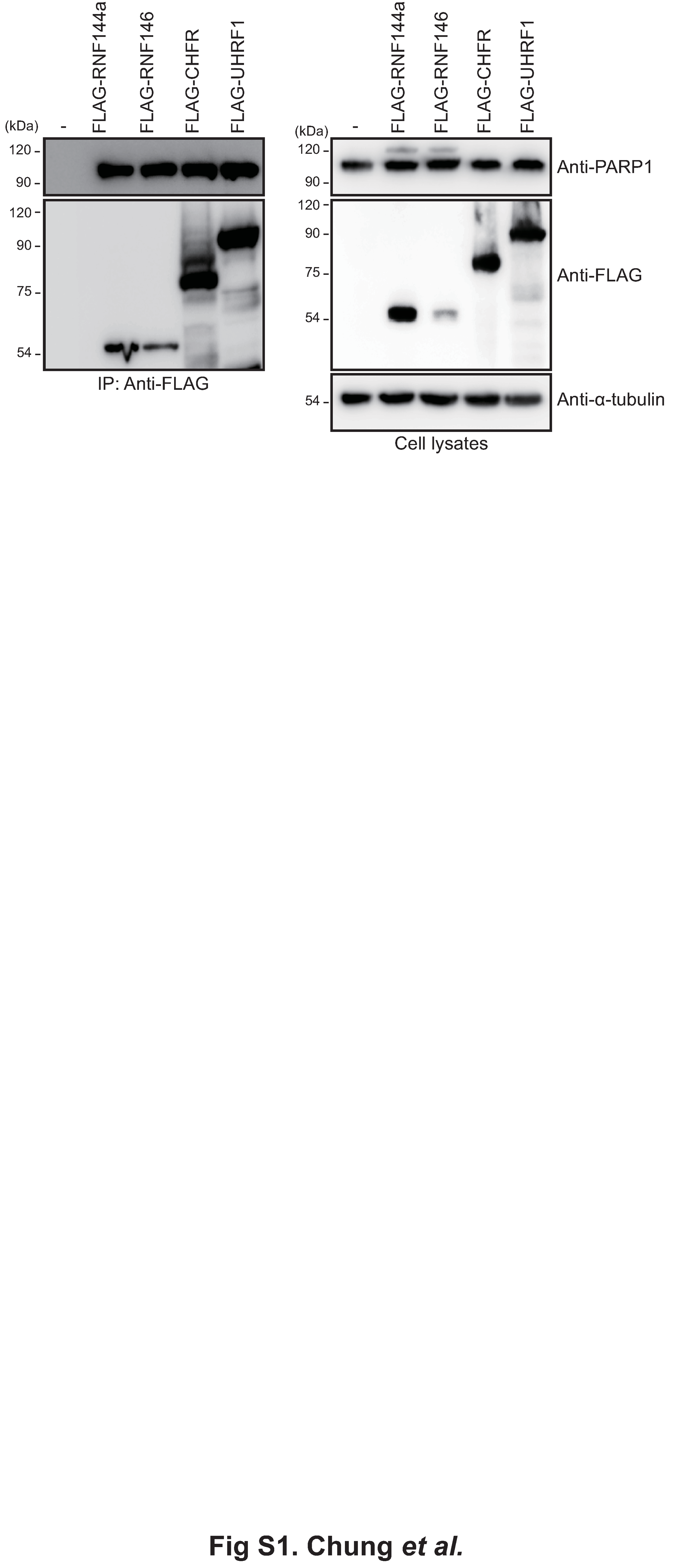

Supplement: S1 Fig — PARP1 interaction with cellular E3 ubiquitin ligases. HEK293T cells were transfected with FLAG-tagged RNF144a, RNF146, CHFR, or UHRF1. The transfected cells were harvested at 48 h post-transfection and subjected to an immunoprecipitation assay with the anti-FLAG antibody. The cell lysates were analyzed by western blotting with the anti-FLAG-M2, anti-PARP1, and anti-α-tubulin antibodies. (TIF) [file ppat.1009261.s001.tif]

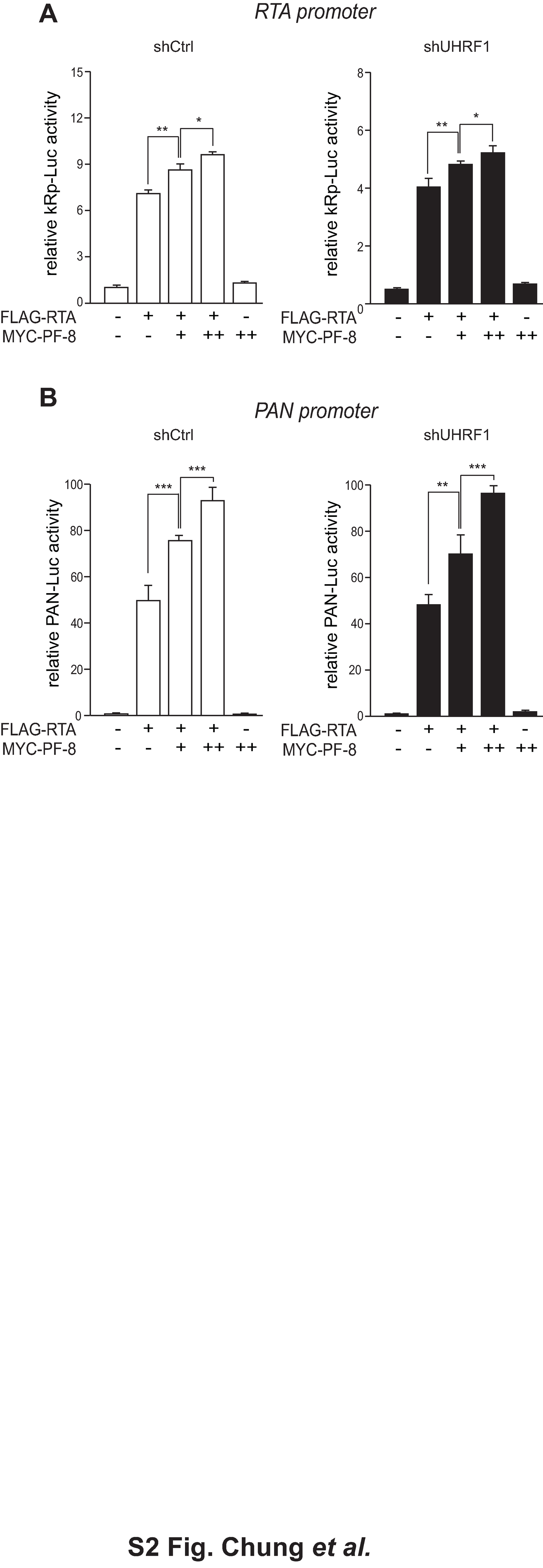

Supplement: S2 Fig — (A and B) Luciferase reporter assays of PF-8 in shUHRF1-transfected cells. The shUHRF1-transfected or shCtrl-transfected HEK293T cells were cotransfected with reporter construct pGL3-kRP-Luc (A) or pGL3-PAN-Luc (B) (300 ng) and MYC-tagged PF-8 (150 or 300 ng) in the presence or absence of the FLAG-tagged RTA expression plasmid (25 ng). The cells were harvested at 48 h post-transfection for luciferase reporter assays. Each transfection was performed in triplicate, and the EGFP-expressing plasmid served as an internal control. Statistical analysis was carried out by Student’s t test (*P < 0.05, **P < 0.01, and ***P < 0.005). (TIF) [file ppat.1009261.s002.tif]

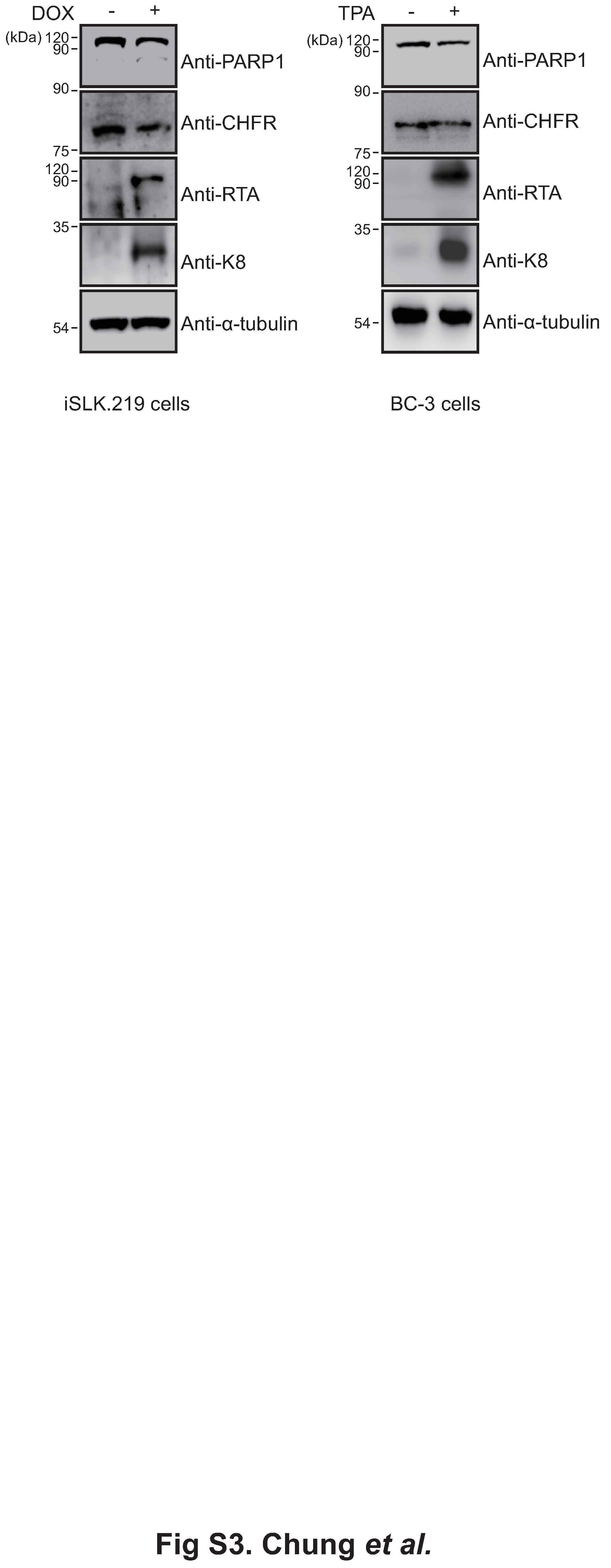

Supplement: S3 Fig — iSLK.219 cells and BC-3 cells latently infected with KSHV were treated with doxycycline (DOX) for 48 h or 12-O-tetradecanoylphorbol-13-acetate (TPA) for 24 h to induce viral reactivation. The cells were harvested and assayed by western blotting with the anti-PARP1, anti-CHFR, anti-RTA, anti-K8, and anti-α-tubulin antibodies. (TIF) [file ppat.1009261.s003.tif]

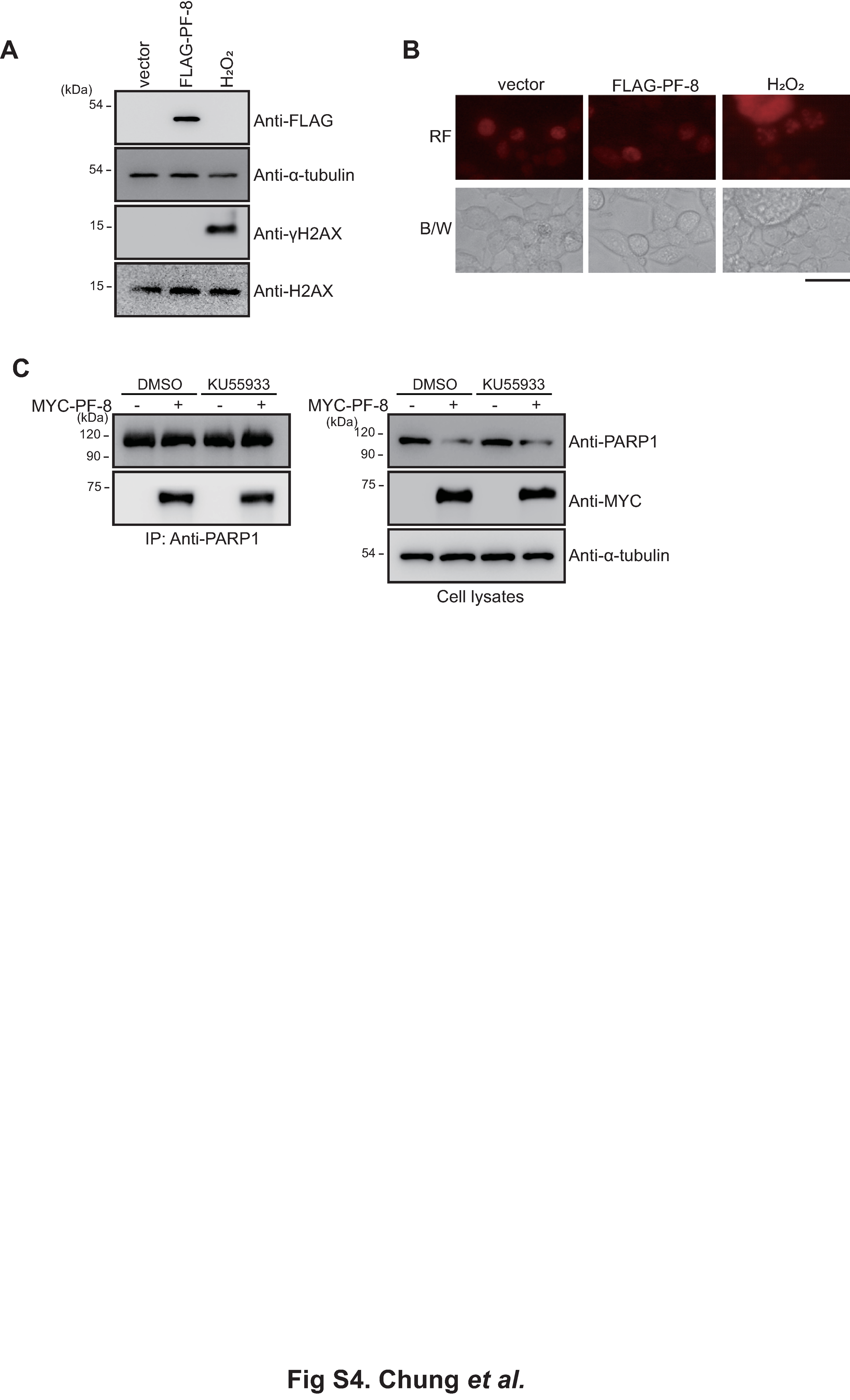

Supplement: S4 Fig — (A) Phosphorylation of H2AX in SLK cells. SLK cells were transduced with a FLAG-tagged PF-8 or control lentiviral vector. As a control, 1 mM H2O2 was treated for 30 min. The cells were harvested and analyzed by western blotting with the anti-γH2AX, H2AX anti-FLAG-M2 and anti-α-tubulin antibodies. (B) 53BP1 recruitment in HEK293T cells. DNA damage reporter HEK293T cells were generated by transducing the cells with a lentiviral vector expressing truncated 53BP1 (amino acids 1220–1711) to Apple fluorescent protein. The cells were transfected with FLAG-tagged PF-8 or treated with 1 mM H2O2 for 30 min. The samples were examined for red-fluorescence under a fluorescence microscope (Leica DM IL LED fluo, Leica). Scale bar, 20 μm. (C) PARP1 degradation and interaction with PF-8 upon ATM kinase inhibitor treatment. HEK293T cells were transfected with MYC-tagged PF-8. After 32 h post-transfection, media were changed and the cells were treated with 10 μM KU55933 for 16 h. The cells were harvested and assayed by IP using the anti-PARP1 antibody. The cell lysates were analyzed by western blotting with the anti-PARP1, anti-MYC, and anti-α-tubulin antibodies. (TIF) [file ppat.1009261.s004.tif]
